# PRC2 direct transfer from G-quadruplex RNA to dsDNA has implications for RNA-binding chromatin modifiers

**DOI:** 10.1073/pnas.2220528120

**Published:** 2023-05-30

**Authors:** Wayne O. Hemphill, Regan Fenske, Anne R. Gooding, Thomas R. Cech

**Affiliations:** ^a^Department of Biochemistry, BioFrontiers Institute, University of Colorado Boulder, Boulder, CO 80309; ^b^HHMI, University of Colorado Boulder, Boulder, CO 80309

**Keywords:** methyltransferase, polynucleotide, nucleosomes, displacement, exchange

## Abstract

The chromatin-modifying enzyme, Polycomb Repressive Complex 2 (PRC2), deposits the H3K27me3 epigenetic mark to negatively regulate expression at numerous target genes, and this activity has been implicated in embryonic development, cell differentiation, and various cancers. A biological role for RNA binding in regulating PRC2 histone methyltransferase activity is generally accepted, but the nature and mechanism of this relationship remains an area of active investigation. Notably, many in vitro studies demonstrate that RNA inhibits PRC2 activity on nucleosomes through mutually antagonistic binding, while some in vivo studies indicate that PRC2’s RNA-binding activity is critical for facilitating its biological function(s). Here we use biochemical, biophysical, and computational approaches to interrogate PRC2’s RNA and DNA-binding kinetics. Our findings demonstrate that PRC2-polynucleotide dissociation rates are dependent on the concentration of free ligand, indicating the potential for direct transfer between nucleic acid ligands without a free-enzyme intermediate. Direct transfer explains the variation in previously reported dissociation kinetics, allows reconciliation of prior in vitro and in vivo studies, and expands the potential mechanisms of RNA-mediated PRC2 regulation. Moreover, simulations indicate that such a direct transfer mechanism could be obligatory for RNA to recruit proteins to chromatin.

## Significance

Studies of PRC2 in vitro indicate that RNA inhibits its histone methyltransferase (HMTase) activity through mutually antagonistic binding with nucleosomes, but some in vivo studies paradoxically suggest that RNA binding is necessary to facilitate its chromatin occupancy and HMTase activity. Our findings unveil a mechanism for direct exchange of RNA and DNA/nucleosome on the PRC2 protein complex, which reconciles these prior findings by allowing RNA regulation of PRC2 to be antagonistic or synergistic depending on RNA–nucleosome proximity. Furthermore, there is an increasing awareness that multiple chromatin-associated proteins exhibit regulatory RNA binding activity, and our findings indicate that this “direct transfer” mechanism may be generally required for RNA recruitment of proteins to chromatin.

PRC2 is a histone methyltransferase (HMTase) that sequentially deposits three methyl groups onto lysine 27 of histone H3 [H3K27me1/2/3; (1–4), reviewed in refs. 5–7], and its activity is crucial for epigenetic silencing during development and cancer (5). How PRC2 is targeted to genetic loci is of considerable interest, given its critical function and abundance of target genes (8). PRC2’s core subunits include the Enhancer of Zeste Homolog 2 (EZH2) catalytic domain, Suppressor of Zeste 12 (SUZ12) scaffold subunit, Embryonic Ectoderm Development (EED) histone tail-binding subunit, and Retinoblastoma-Binding Protein 4 (RBBP4) histone chaperone subunit, and it has additional accessory subunits that define the PRC2.1 and PRC2.2 subtypes and differentially regulate its activity [(9–11), reviewed in ref. 5]. PRC2 binds numerous long noncoding RNAs (lncRNAs) and pre-mRNAs in cell nuclei, and this RNA binding is believed to regulate PRC2’s HMTase activity (12–15). Furthermore, biochemical studies have demonstrated that PRC2 has specificity for G-tracts and G-quadruplex (G4) RNA structures (16), which are ubiquitous in the human transcriptome, consistent with its widespread RNA binding in cells.

The nature and mechanism(s) of PRC2 regulation by RNA remain quite controversial. While some studies have proposed a role for RNA in PRC2 recruitment to chromatin (13, 17), others have suggested roles in PRC2 eviction from chromatin and/or inhibition of PRC2 catalytic activity (18–22), and these ideas are not mutually exclusive. Biochemical experiments have convincingly demonstrated that RNA antagonizes PRC2 HMTase activity (18, 20, 22), and that this is mediated by competitive binding with nucleosomes (19, 20). On the other hand, a recent work has demonstrated that the PRC2-RNA interaction is critical in vivo for maintaining H3K27me3 levels and chromatin occupancy at PRC2 target genes in induced pluripotent stem cells (23). It is prudent to note that the biochemical studies have utilized RNA and nucleosomes in free solution, which is not representative of the chromatin-associated nascent RNA suspected to regulate PRC2 activity in vivo (18, 19, 21, 22). Furthermore, the role(s) of RNA in PRC2 activity could be contextual to chromatin architecture, available PRC2 accessory subunits and protein partners, competing RNA-binding proteins, and/or post-translational modifications. Thus, prior biochemical studies may lack considerations relevant to in vivo function. The direct evidence for a mechanism that can reconcile RNA antagonizing PRC2’s nucleosome binding and HMTase activity in vitro with RNA-mediated PRC2 recruitment in vivo has yet to be reported.

Herein, we measure the kinetics of human PRC2’s RNA and DNA binding using biochemical, biophysical, and computational methods. Our findings unexpectedly reveal that PRC2 has the intrinsic ability to exchange one nucleic acid for another without completely dissociating from the first nucleic acid. Such mechanisms have been well-studied for homo-multimeric DNA-binding proteins like lac repressor (24), *E. coli* catabolite activator protein (CAP) (25), SSB (26), and recA (27) and for the hexameric RNA-binding protein Hfq (28). Historically, this phenomenon has been variously identified as “concentration-dependent dissociation,” “direct transfer” (25–27), “facilitated exchange” (29), or “active exchange” (28), and it is related to the sister phenomena of protein movement along DNA (30–34) and “facilitated dissociation” (35, 36). The proteins in these cases are homo-oligomeric, and as others have noted (37), their multiple ligand-binding sites likely facilitate direct transfer by providing a foothold for a second ligand before it displaces a previously bound ligand.

We propose that PRC2’s ability to directly transfer from one nucleic acid to another may reconcile the disparate eviction versus recruitment models of previous studies. Furthermore, binding to nascent RNA has been suggested as a general strategy by which transcription factors and other DNA-binding proteins are maintained at high local concentrations for recruitment to target genes. Our findings indicate that this model may be feasible only if the protein can directly transfer from RNA to DNA without dissociation, suggesting direct transfer capabilities may have general relevance.

## Results

### PRC2 Exhibits Direct Transfer Between G4 RNA and dsDNA.

**Fig. 1.**
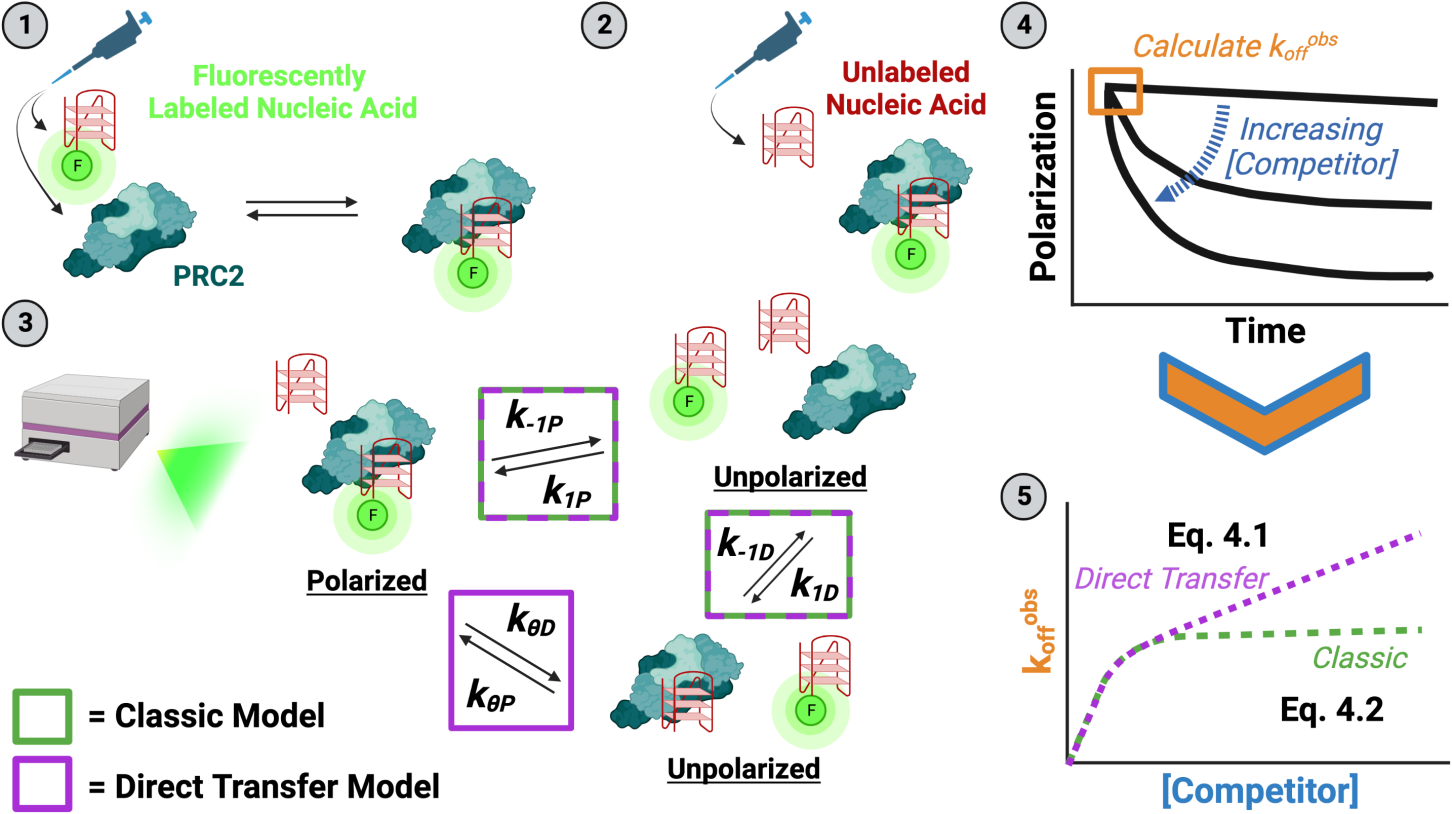
Experiment and analysis strategy to measure direct transfer kinetics (FPCD Experiments). (1) The minimum amount of PRC2 required for saturated binding is mixed with a trace amount of fluorescently labeled nucleic acid (ligand), then incubated (at 4/25 °C) until thermal and reaction equilibrium. (2) Various concentrations of unlabeled nucleic acid (competitor) are added to the preformed complex to initiate reactions (at 25 °C). (3) The time-course reactions are immediately monitored by fluorescence polarization in a microplate reader (at 25 °C). Potential complexes with their polarization states are shown, and they are labeled with rate constants describing inter-complex transitions. Rate constants associated with a classic competition model are indicated by green boxes, and those additionally necessary for a direct transfer model are indicated by a purple box. The intercomplex transition solely associated with the direct transfer model has an implied unstable ternary complex intermediate. The system of differential equations describing these reactions is given by *SI Appendix*, Eq. **S1**. (4) Polarization signals are normalized to the range in polarization signal across all competitor concentrations to give proportion of initial complex remaining. Normalized polarization signals are plotted versus time and fit with one-phase exponential decay regression (*SI Appendix*, Eq. **S3.1**). (5) The regression initial slopes (k_off_^obs^; *SI Appendix*, Eq. **S3.2**) are plotted versus competitor concentration and regressed with custom equations describing the classic competition (*SI Appendix*, Eq. **S4.2**) and direct transfer (*SI Appendix*, Eq. **S4.1**) models to determine rate constant values. Model fits are compared with the Bayesian Information Criterion (BIC) to determine the appropriate model.

**Fig. 2.**
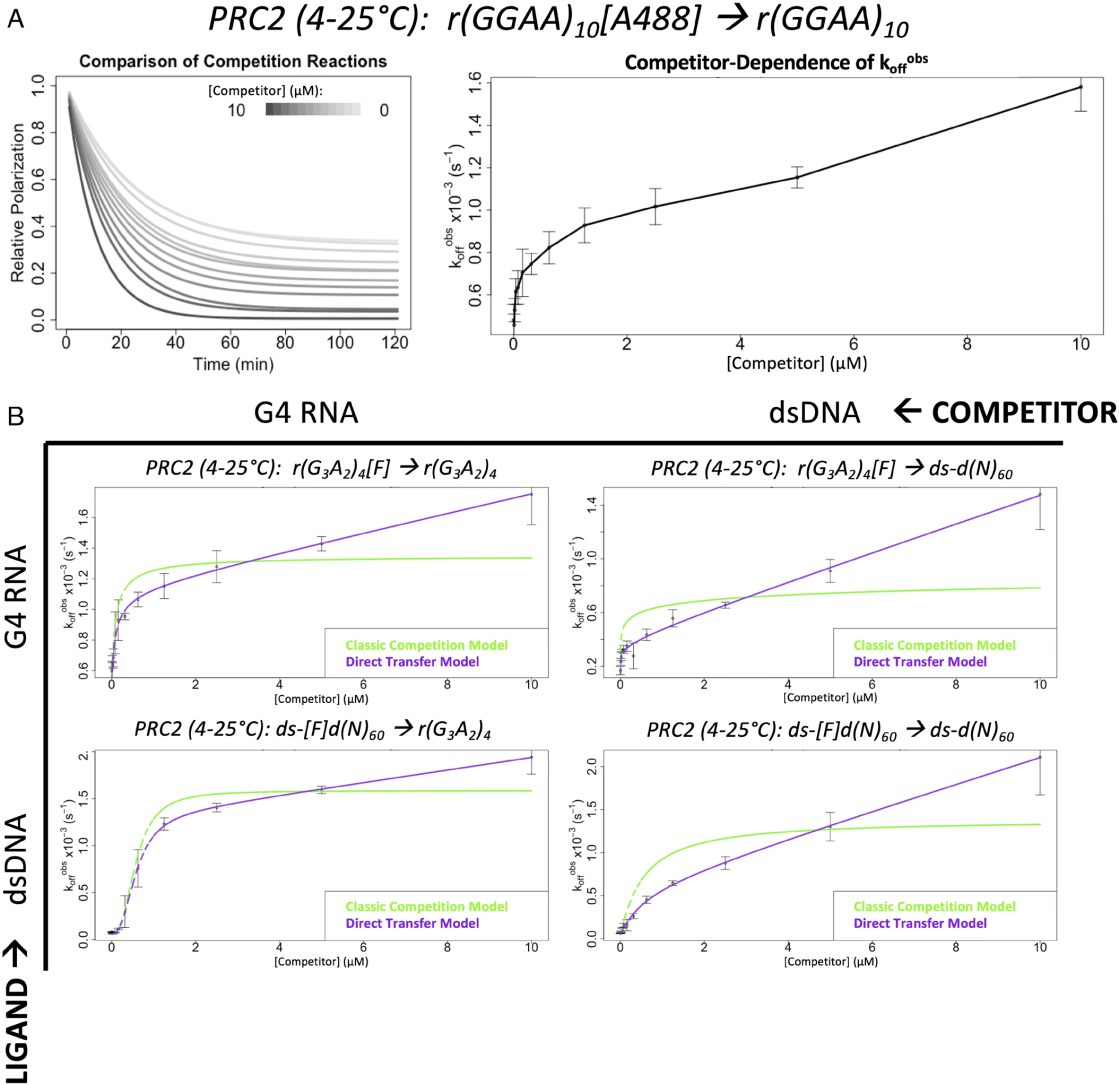
PRC2 exhibits direct transfer kinetics for G4 RNA and dsDNA. FPCD experiments (Fig. 1) were performed with the Wang et al. and Long et al. ligand/competitor over a range of competitor concentrations (panel *A*) and to measure direct transfer kinetics for every ligand–competitor combination of a G4 RNA and 60-bp dsDNA (panel *B*). Data are from representative experiments (of n ≥ 3), where error bars indicate mean ± SD for four technical replicates. Rate constant values from regression can be found in Table 1, additional nomenclature definitions are in *SI Appendix*, Table S1, and polynucleotide species definitions are in *SI Appendix, *Table S2. (*A*) Exponential regression fit lines from each condition (left plot), alongside the observed initial dissociation rates (k_off_^obs^, see *SI Appendix*, Eq. **S3.2**) as a function of competitor concentration (right plot). Solid line in right plot is a visual aid connecting data means. Raw data are shown in *SI Appendix*, Fig. S1. (*B*) Experiments were performed in BB_10_ buffer. Isotherm, carrier nucleic acid, and fluorophore controls for the RNA-RNA competition experiment (*Top Left*) can be found in *SI Appendix*, Fig. S2. Analogous studies with a 50-bp dsDNA can be found in *SI Appendix*, Fig. S3.

Two prior studies from our group (19, 23) determined the dissociation rate constant for a G4 RNA species from PRC2, but they obtained significantly different values despite nearly identical methodologies. One of the few methodological distinctions between these two studies was the concentration of unlabeled competitor RNA used to prevent the rebinding of labeled RNA once it dissociated from PRC2. Long et al. and Wang et al. used a 200- and 2,000-fold excess of competitor RNA over RNA ligand, respectively, both of which should have been sufficient to totally prevent ligand rebinding. We used fluorescence polarization (FP)-based competitive dissociation (FPCD) experiments (Fig. 1) to replicate the Wang/Long studies across a range of competitor RNA concentrations. Unexpectedly, the observed dissociation rate (k_off_^obs^) of PRC2 and G4 RNA did not plateau at excess concentrations of competitor, but it instead continued to increase linearly in a competitor concentration-dependent manner (Fig. 2*A* and *SI Appendix*, Fig. S1). This result is consistent with the incoming competitor being able to displace the initially bound ligand without free PRC2 as an intermediate, i.e., direct transfer of PRC2 between ligand and competitor (26). Our data are consistent with both the Wang et al. and Long et al. findings given the respective competitor concentrations they used.

Since our initial experiments utilized an RNA with 10 G-tracts that could form G4s heterogeneously, we tested a simpler RNA sequence containing only four G-tracts and found that it also exhibited direct transfer kinetics (*SI Appendix*, Fig. S2*A*). Next, we repeated this experiment at constant room temperature (*SI Appendix*, Fig. S2*B*) to interrogate temperature-dependent effects. Then, we repeated the experiment using a carrier poly(A) RNA that does not bind PRC2 to keep total RNA concentration constant in the reactions (*SI Appendix*, Fig. S2*C*), so that any nonspecific polynucleotide concentration-dependent phenomena (e.g., electrostatic effects) could be ruled out as artifactual explanations. Finally, we repeated the experiment with a different fluorescent label on the ligand molecule (*SI Appendix*, Fig. S2*D*) to interrogate interactions with the fluorophore. The data from all experiments were well fit by a regression model allowing direct transfer kinetics but poorly fit by a classic model of competition. We conclude that PRC2 exhibits direct transfer between G4 RNAs.

Of particular biological relevance is PRC2’s potential for direct transfer between RNA and chromatin. Prior studies indicate that PRC2 affinity for nucleosomes is entirely mediated by exposed nucleosome linker DNA (19), suggesting comparable-length dsDNA species should be representative of PRC2’s nucleosome binding activity. Thus, we performed FPCD experiments with our simple G4 RNA and a 60-bp dsDNA, using all possible ligand–competitor combinations (Fig. 2*B*). Notably, our results indicate that direct transfer occurs between all species (Table 1). Experiments with a 50-bp dsDNA species produced qualitatively similar results (*SI Appendix*, Fig. S3). We also note that prior reports of PRC2 dsDNA and G4 RNA binding affinities (13, 16, 19, 23) are consistent with our corresponding values in Table 1.

**Table 1.** Rate constants for a variety of protein–ligand interactions

Ligand	Buffer	T (°C)	K_dP_^app^ (nM)	Competitor	k_−1P_ (s^−1^)	k_θD_ (M^−1^s^−1^)
r(GGAA)_10_[A488]	BB_25_	25	^*^78 ± 12	r(GGAA)_10_	^*^,^†^n.d.	^*^,^†^n.d.
		4 to 25	n.d.	r(GGAA)_10_	^*^5.2 ± 0.5 (×10^−4^)	^*^79 ± 6.0
r(G_3_A_2_)_4_[F]		BB_10_	25	^‡^2.3 ± 0.35	ds-d(N)_50_	^||^1.4 ± 1.1 (×10^−3^)	^||^91 ± 31
	4 to 25		n.d.	r(G_3_A_2_)_4_	8.3 ± 1.6 (×10^−4^)	30 ± 13
				ds-d(N)_60_	^||^4.5 ± 0.15 (×10^−4^)	^||^100 ± 19
				BB_25_	25	4.4 ± 0.34	r(G_3_A_2_)_4_	1.7 ± 0.60 (×10^−3^)	660 ± 130
		4 to 25			n.d.	r(G_3_A_2_)_4_	5.6 ± 0.50 (×10^−4^)	47 ± 17
	^§^r(G_3_A_2_)_4_ | C_1_					^¶^4.7 × 10^−4^	^¶^73
	BB_100_		25			^‡^1.4 ± 0.15	–	–	–
	BB_200_		25	6.0 ± 0.93		–	–	–
r(G_3_A_2_)_4_[A488]	BB_25_	25	8.2 ± 0.69	–		–	–
		4 to 25	n.d.	r(G_3_A_2_)_4_	8.8 ± 0.32 (×10^−4^)	59 ± 20
ds-[F]d(N)_60_		BB_10_	25	5.1 ± 0.60	r(G_3_A_2_)_4_	2.4 ± 0.25 (×10^−3^)	170 ± 41
	ds-d(N)_60_				^¶^9.1 × 10^−5^	^¶^260
	4 to 25				n.d.	r(G_3_A_2_)_4_	1.2 ± 0.062 (×10^−3^)	67 ± 8.8
						ds-d(N)_60_	5.3 ± 2.3 (×10^−4^)	150 ± 21
			BB_25_			25	82 ± 13	–	–	–
		BB_100_	25	170 ± 15		–	–	–
	BB_200_	25	^#^n/a	–		–	–
ds-d(N)_50_[F]	BB_10_	25	5.0 ± 0.46	r(G_3_A_2_)_4_	2.5 ± 0.11 (×10^−3^)	170 ± 8.2
				ds-d(N)_50_	7.6 ± 0.75 (×10^−4^)	210 ± 91
				4 to 25	n.d.	ds-d(N)_50_	^¶^2.8 × 10^−4^	^¶^340
				BB_25_	25	390 ± 31	–	–	–

^*^Experiments used PRC2_5m_ (somatic AEBP2 isoform), not PRC2_5me_ (embryonic AEBP2 isoform).

^†^Dissociation completed during initiation-measurement delay (~90 s; λ ≥ 3.3 × 10^−2^ s^−1^).

^‡^Experiment used [Ligand] ≥ 2× K_dP_^app^; it’s possible that K_d_ < K_d_^app^.

^§^Total polynucleotide concentration was kept constant by serially diluting competitor in a carrier nucleic acid; C_1_ = r(A)_20_.

^¶^Value from single experiment.

^#^Binding too weak to obtain K_d_^app^ (> 1 µM).

^||^Weak competitor—manual baseline (from binding curve) used for regression calculations.

Fluorescence polarization-based methodology (Fig. 1 and *Materials and Methods*) was used to determine the apparent equilibrium dissociation constants (K_dP_^app^) (in the absence of competitor), intrinsic dissociation rate constants (k_−1P_), and direct transfer rate constants (k_θD_). Values indicate mean ± SD for at least three independent experiments. K_d_^app^ are from regression with a standard (non-quadratic, non-Hill) binding equation (*SI Appendix*, Eq. **S2**). Numerical subscript of buffers refers to their variable concentration of salt, and specific buffer definitions can be found in *Materials and Methods*. Additional nomenclature definition is provided in *SI Appendix*, Table S1, and polynucleotide sequences are defined in *SI Appendix, *Table S2. n.d. = not determined; n/a = not applicable.

### PRC2 May Have Additional Electrostatic Contacts with dsDNA Not Utilized for G4 RNA.

**Fig. 3.**
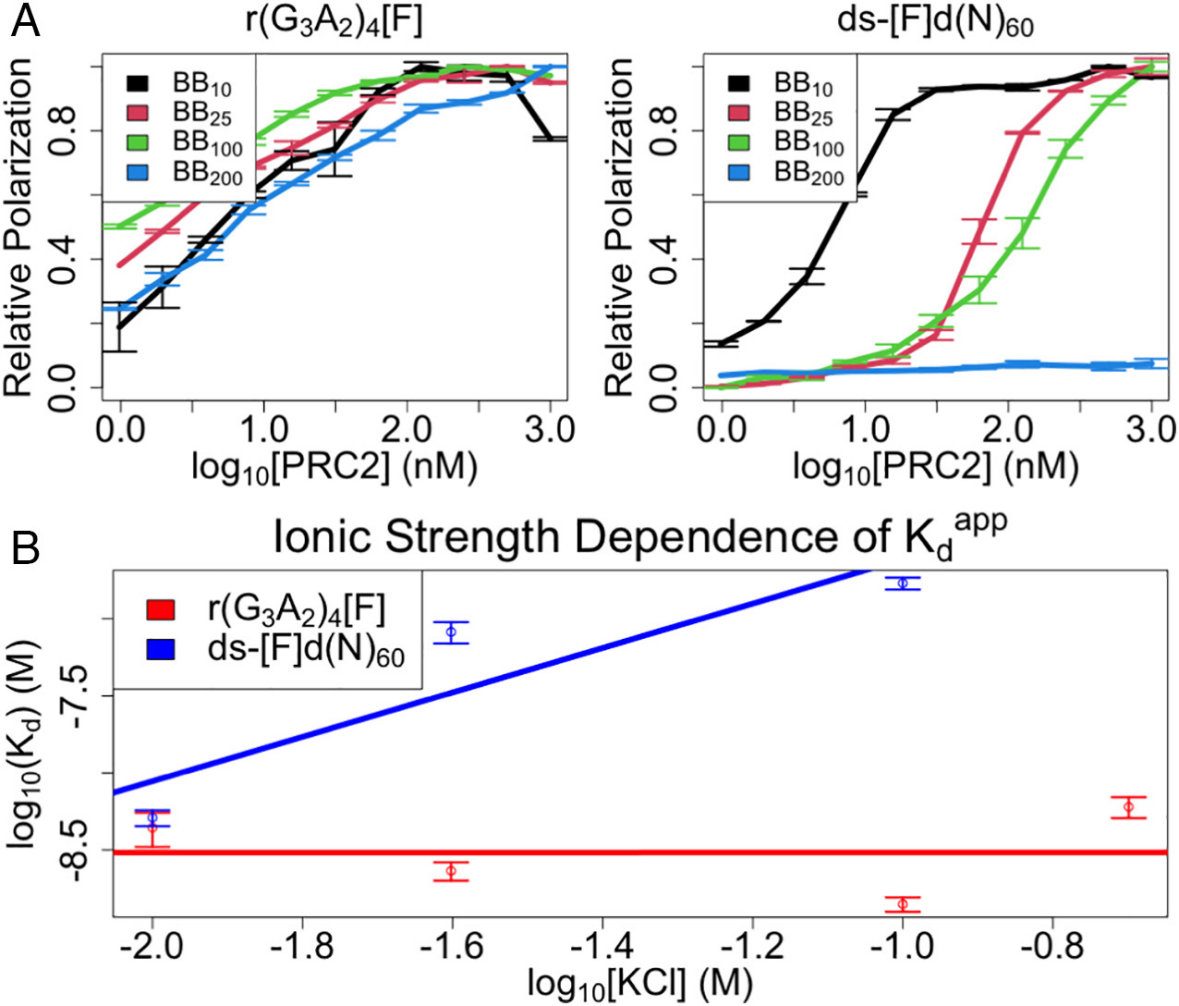
Ionic interactions contribute to PRC2’s dsDNA but not G4 RNA affinities. FP-based equilibrium binding experiments (*Materials and Methods*) were carried out under various salt concentrations (BB_X_ = X mM KCl) for a G4 RNA and 60-bp dsDNA ligand (no competitor present). Kinetic constant values from regression can be found in Table 1, additional nomenclature definitions are in *SI Appendix, *Table S1, and polynucleotide species definitions are in *SI Appendix, *Table S2. (*A*) Binding curves for indicated PRC2 ligands. Curves are composites of three experiments with four replicates each, where error bars indicate mean ± SD. Solid lines are visual aids connecting the data points. (*B*) Affinity versus ionic strength plots with K_d_^app^ values from regression of data in panel *A*. Data are composites of all experiments in panel *A*, where error bars indicate mean ± SD. Solid lines are from linear regression of data on the logarithmic axes shown. Regression values can be found in *Materials and Methods* or the corresponding text.

Prior studies indicate that G4 RNA and dsDNA binding to PRC2 are mutually antagonistic (i.e., competitive) (19, 20), which may suggest competition for shared protein-polynucleotide contacts. However, it is not clear to what extent the PRC2 binding surfaces for RNA and DNA may have some unique contacts. If such unique interactions had an electrostatic component, they might be revealed by differential salt sensitivity. We therefore used FP to determine K_d_^app^ for G4 RNA and dsDNA at a range of salt concentrations. The experiments demonstrated a much greater influence of ionic strength on PRC2’s binding affinity for dsDNA than for G4 RNA (Fig. 3*A*). Linear regression of log(K_d_^app^) versus log([KCl]) plots (Fig. 3*B*) suggests that more salt bridges mediate PRC2 binding to dsDNA (m ≈ 1.4 ± 0.68) versus G4 RNA (m ≈ 0 ± 0.34) (38), which is consistent with the previous conclusion that the PRC2-RNA interaction is not primarily electrostatic (39). It is prudent to note, however, that ionic strength dependence could also be affected by other properties like nucleic acid conformation and divalent ion concentration (38, 40). Thus, a simplistic interpretation of these data suggests that PRC2 has additional ionic contacts with dsDNA that are not utilized during its binding to G4 RNA.

### Modeling Suggests the PRC2 Direct Transfer Mechanism Allows RNA-Mediated Recruitment to Nucleosomes.

**Fig. 4.**
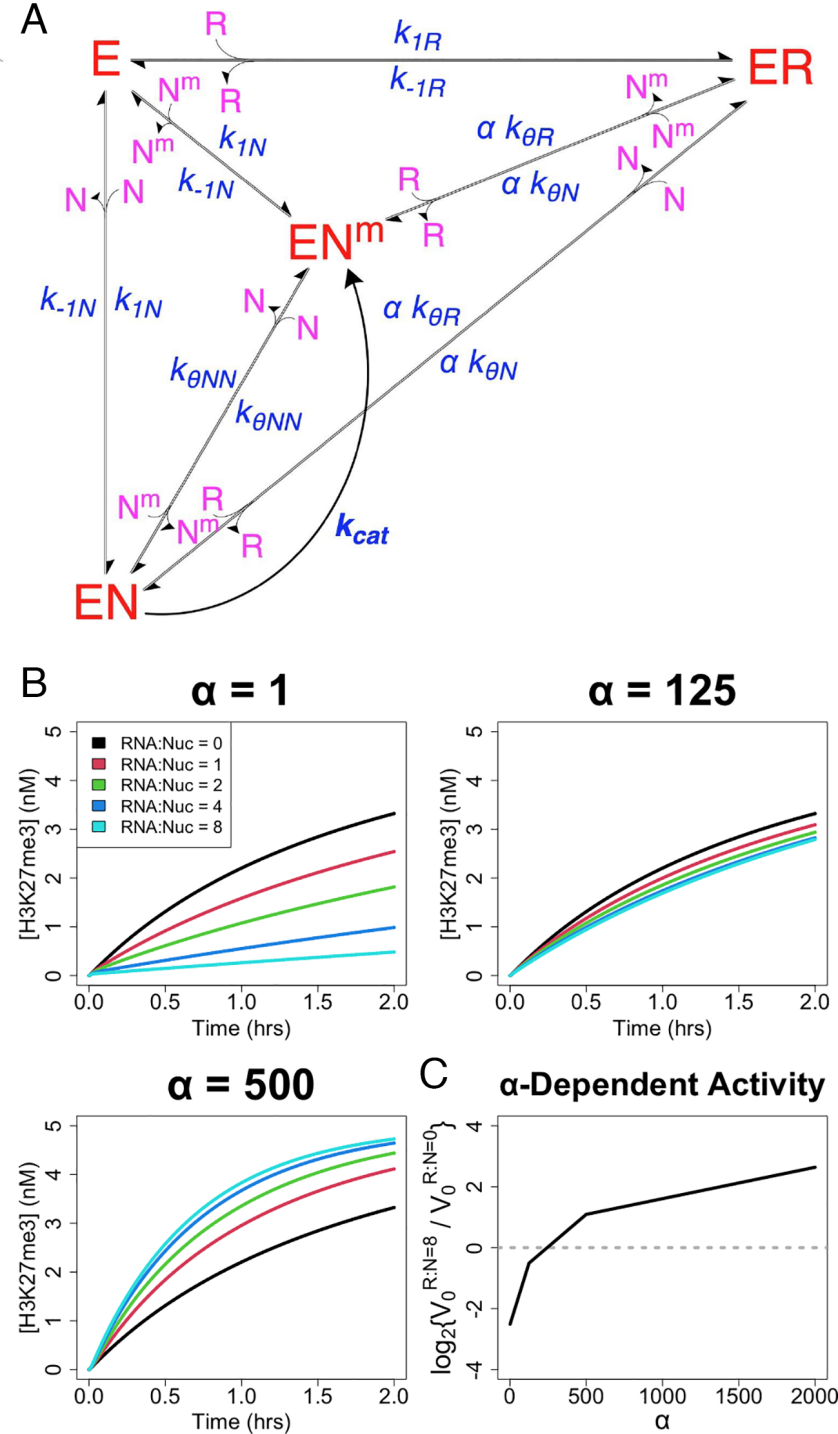
Direct transfer allows RNA to boost PRC2 HMTase activity. (*A*) Reaction scheme of PRC2-like protein (E) binding of RNA (R) and nucleosomes (N) with catalytic activity on nucleosomes (N^m^), where conjugates are complexes of the respective reactants. Major protein states are shown in red, additional reactants in purple, and rate constants and tuning parameters in blue. For rate constants, k_1_ is for association, k_−1_ is for dissociation, k_θ_ is for direct transfer, and k_cat_ is for catalysis. The α tuning parameter is included for any protein complex transitions where RNA–nucleosome direct transfer is possible. It is an adjustment of effective molarity for direct transfer reactions, meant to account for the spatial proximity of nascent RNA and nucleosome DNA (e.g., nascent RNA), but it has a complex relationship with other factors that warrants qualitative interpretation (see corresponding text). Specific nomenclature definitions are in *SI Appendix*, Table S1. These reactions are described by the system of differential equations, *SI Appendix*, Eq. **S5** . Inter-complex transitions defined by the k_θ_ rate constants are like those shown in Fig. 1, and their removal collapses this scheme to a classic model. (*B*) Reactions were simulated using *SI Appendix*, Eq. **S5** for the scheme (panel *A*) to monitor rate of nucleosome methylation (H3K27me3) over time under varying RNA–nucleosome molar ratios (RNA:Nuc), direct transfer effective molarity adjustments (α), and protein concentrations. Black curves represent HMTase time-course reactions in the absence of RNA, and the colored lines represent the effect of increasing RNA concentrations. For simulations, k_−1_, k_θ_, and K_d_ values were taken from Table 1, k_cat_ was taken from prior PRC2 literature, [N_T_] = 5 nM, [E_T_] = 0.1–2 × K_dN_, and other parameter values are indicated; explicit values are provided in *Materials and Methods*. Limited data from a single protein concentration (2 × K_dN_) are shown, but the full data set is provided in *SI Appendix*, Fig. S4. (*C*) HMTase activity rate data were used to calculate the relative initial rates (V_0_) for reactions with 8:1 versus 0:1 RNA–nucleosome molar ratios (R:N), across a range of α values. Data used were the same as for panel *B*. Dotted line is a visual aid for when RNA concentration has no effect on initial reaction rate.

Prior studies indicate that RNA inhibits PRC2’s nucleosome DNA binding and HMTase activity (13, 19, 20), while others paradoxically suggest that RNA facilitates PRC2 chromatin occupancy and H3K27me3 deposition (22, 23). To interrogate whether PRC2 direct transfer might reconcile these views, we constructed a reaction scheme of PRC2’s proposed biochemical activity (Fig. 4A). This scheme accounts for classic PRC2 (E) binding to (k_1_) and dissociation from (k_−1_) RNA (R) and nucleosome DNA (N), PRC2 mutually antagonistic binding to RNA and nucleosome, the catalytic deficiency of RNA-bound PRC2 (19, 20), and PRC2 catalytic (k_cat_) methylation of nucleosomes (N^m^). In addition, the model includes the direct transfer reactions (k_θ_) demonstrated by our present studies (Fig. 2). To account for the in vivo proximity between nascent RNA and chromatin, we incorporate a simple tuning parameter (α) for the effective molarity between RNA and nucleosome in direct transfer reactions. We note that while effective molarity for these reactions should increase when RNA–nucleosome proximity is increased by tethering (e.g., nascent RNA), the degree of increase would have a complex relationship with other factors such as nascent RNA length, caging effects in condensates, and/or the relative prevalence of free versus nascent RNA. Consequently, the parameter’s effects are best interpreted semi-quantitatively (if not qualitatively), and quantitative conclusions about how RNA length and other factors affect protein activity are outside the scope of our studies.

We simulated reactions under this scheme using our empirically determined rate constants for association, dissociation, and direct transfer events (Table 1) and using the previously reported rate constant for EZH2 (PRC2 catalytic subunit) methylation of nucleosomes (41). The results (Fig. 4*B* and *SI Appendix*, Fig. S4) indicate that RNA should be antagonistic to PRC2 HMTase activity in free solution (α = 1), as observed experimentally. However, RNA should eventually become synergistic as the RNA–nucleosome effective molarity in direct transfer events increases (e.g., α = 500).

As expected, all RNA effects on PRC2 HMTase activity are ablated if the PRC2-RNA complex is unstable (k_−1R_ × 10^9^) (*SI Appendix*, Fig. S5). Notably, the ability of RNA to boost PRC2 HMTase activity is completely ablated if direct transfer is ablated (k_θ_ = 0) (*SI Appendix*, Fig. S6). Overall, these data suggest that PRC2’s mutually antagonistic RNA and nucleosome binding could be reconciled with RNA-mediated recruitment of PRC2 to chromatin under some conditions, but only if PRC2 can direct transfer from RNA to nucleosomes.

### Direct Transfer May Be Generally Required for RNA Recruitment of Chromatin-Associated Proteins.

**Fig. 5.**
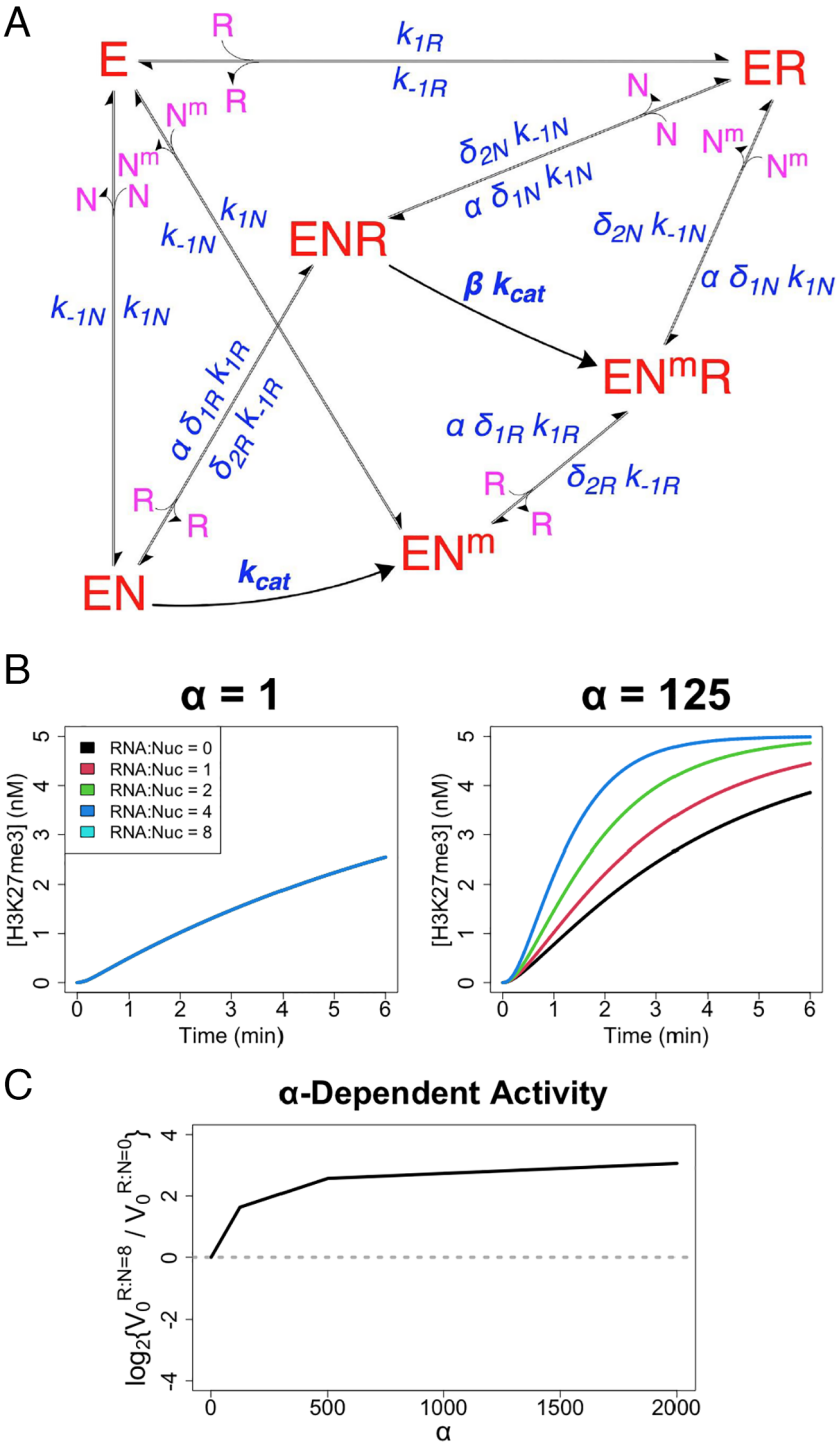
Stable RNA and nucleosome cobinding could boost a protein’s activity. (*A*) Reaction scheme of a protein (E) binding RNA (R) and nucleosomes (N) independently, with the potential for catalytic activity on nucleosomes (N^m^), where conjugates are complexes of the respective reactants. Major protein states are shown in red, additional reactants in purple, and rate constants and tuning parameters in blue. For rate constants, k_1_ is for association, k_−1_ for dissociation, and k_cat_ is for catalysis. The α tuning parameter is included for any protein complex transitions where ternary complex formation from a bimolecular complex is possible. It is an adjustment of effective molarity for direct transfer reactions, meant to account for the spatial proximity of nascent RNA and nucleosome DNA (e.g., nascent RNA), but it has a complex relationship with other factors that warrants qualitative interpretation (see corresponding text). The β tuning parameter is effect of bound RNA on catalytic activity. The δ tuning parameters are the effects of bound RNA/nucleosome on ternary nucleosome/RNA binding or dissociation. Specific nomenclature definitions are in *SI Appendix*, Table. S1. (*B*) Reactions were simulated using *SI Appendix*, Eq. **S6** for the scheme (panel *A*) to monitor rate of nucleosome methylation (H3K27me3) over time under varying RNA–nucleosome molar ratios (RNA:Nuc) and effective molarity adjustments (α). Black curves represent HMTase time-course reactions in the absence of RNA, and the colored lines represent the effect of increasing RNA concentrations. For α = 1, all lines overlap. For simulations, K_d_ values were taken from Sigova et al, k_cat_ was set to the value from PRC2 literature, [N_T_] = 5 nM, [E_T_] = 0.1−2 × K_dN_, β = 0−1, δ = 1, and other parameter values are indicated; explicit values are provided in *Materials and Methods*. Limited data from a single protein concentration (0.125 × K_dN_) and β value (β = 1) are shown, but the full data set is provided in *SI Appendix*, Fig. S7. (*C*) HMTase activity rate data were used to calculate the relative initial rates (V_0_) for reactions with 8:1 versus 0:1 RNA–nucleosome molar ratios (R:N), across a range of α values. Data used were the same as for panel *B*. Dotted line indicates RNA concentration having no effect on initial reaction rate.

In the case of PRC2 and some other chromatin-associated proteins, RNA and nucleosomal DNA bind mutually antagonistically (i.e., competitively). However, other proteins can stably bind both RNA and chromatin simultaneously. For example, the transcription factor Yin Yang 1 (YY1) binds DNA and RNA independently, and Sigova et al. (42) proposed that its RNA binding keeps YY1 trapped near its DNA binding sites to help recruit it to chromatin DNA. To interrogate this alternative situation of simultaneous binding, we designed a reaction scheme for a hypothetical HMTase enzyme with independent RNA and nucleosome binding activity (Fig. 5*A*). This scheme accounts for classic protein (E) binding to (k_1_) and dissociation from (k_−1_) RNA (R) and nucleosome DNA (N) and the catalytic (k_cat_) methylation of nucleosomes (N^m^). It also utilizes the α tuning parameter for effective molarity (with the same caveats as for Fig. 4*A*), which in this case applies to ternary complex formation from bimolecular complexes. In addition, the scheme accounts for RNA-mediated suppression of catalysis (β) and potential interplay between nucleosome and RNA binding in the context of ternary complex formation (δ_1_) and dissociation (δ_2_).

We simulated reactions under this scheme using kinetic constants for RNA and DNA binding that are consistent with those reported by Sigova et al. for YY1 (42) and using the same methylation rate constant as for PRC2. As expected, our results (Fig. 5*B*) support RNA concentration having no effect on activity in free solution (α = 1) when RNA binding is independent of nucleosome binding (δ = 1) and does not affect catalysis (β = 1). In contrast, under the same conditions the simulations show that RNA concentration facilitates catalytic activity as the effective molarity of ternary complex-forming reactions (e.g., due to RNA–nucleosome proximity) increases (α > 1). However, this synergy is easily ablated by even minor RNA-mediated catalytic suppression (β < 1) (*SI Appendix*, Fig. S7). Importantly, this synergy is completely dependent on formation of the stable ternary complex, and preventing its formation (δ_1_ = 0) ablates RNA-dependent increases in activity (*SI Appendix*, Fig. S8). Thus, direct translocation between RNA and nucleosome DNA without a free-enzyme intermediate seems necessary to improve activity rate for the alternative situation of independent RNA and nucleosome binding.

These data address chromatin binders with catalytic activity, but RNA-binding transcription factors (RBTFs) like YY1 have biological activity that is not catalytic in nature. To interrogate the relevance of our findings to such RBTFs, we eliminated catalytic activity (k_cat_ = 0) from the prior reaction scheme (Fig. 5*A*) and monitored nucleosome binding. Our results suggest RNA could improve RBTF activity at high effective molarity for ternary complex-forming reactions (α > 1) without any detrimental effects in free solution (α = 1) (*SI Appendix*, Fig. S9*A*). However, this is dependent on an RBTF’s ability to function on its nucleosome target with RNA co-bound (*SI Appendix*, Fig. S9*B*) and on formation of the ternary complex (*SI Appendix*, Fig. S9*C*). Consequently, translocation without a free protein intermediate seems necessary for RNA-mediated facilitation of activity for proteins with independent RNA and nucleosome binding, independent of whether the protein acts catalytically or simply by binding DNA.

These findings indicate that competitive (PRC2-like; Fig. 4*A*) and independent (YY1-like; Fig. 5*A*) RNA and nucleosome binding systems can both have RNA-mediated facilitation of their activity, and that this facilitation is dependent on the ability to translocate between RNA and nucleosomes without a free-protein intermediate. While PRC2 would accomplish this through direct transfer, independent binding systems accomplish this through a stable ternary complex. However, while our PRC2 reaction scheme for direct transfer events (Fig. 4*A*) doesn’t explicitly identify a ternary complex (Fig. 5*A*), the existence of an unstable ternary complex intermediate is still implied. Indeed, making the ternary complex for an HMTase with independent binding (Fig. 5*A*) a million-fold less stable (δ_1_ = 10^−1^, δ_2_ = 10^5^) allows RNA facilitation of activity (*SI Appendix*, Fig. S10). Interestingly, non-catalytic independent binders (i.e., RBTFs) have their RNA-mediated effects ablated by relatively minor destabilization (δ_1_ = 1^−1^, δ_2_ = 10^2^) of the ternary complex if there is no bias between ligands (δ_2N_ = δ_2R_) (*SI Appendix*, Fig. S9*D*). However, a million-fold less stable ternary complex still allows RNA-mediated recruitment (*SI Appendix*, Fig. S9*E*) or inhibition (*SI Appendix*, Fig. S9*F*) if the RBTF has a bias toward RNA (δ_1_ = 10^−1^, δ_2N_ = 10^4^, δ_2R_ = 10^6^) or nucleosome (δ_1_ = 10^−1^, δ_2N_ = 10^6^, δ_2R_ = 10^4^) dissociation from the ternary complex, respectively. Collectively these data suggest that the seemingly distinct PRC2-like and Sigova et al. models for RNA-mediated recruitment to chromatin both rely on translocation between RNA and nucleosome DNA through a ternary complex intermediate, and they differ only in the stability of their ternary complexes (i.e., the lifetime of the ternary intermediate). Thus, some form of direct transfer may be generally necessary for RNA-binding chromatin-associated proteins to have their functions on chromatin facilitated by RNA.

Our data suggest that direct transfer creates a synergistic relationship between RNA concentration and protein activity, but only if the α value (i.e., RNA–nucleosome proximity) is high, implying that direct transfer alone is insufficient for RNA-mediated facilitation of protein function. We next consider whether RNA–nucleosome proximity could allow RNA-mediated protein recruitment to chromatin for strictly exclusive binders (i.e., no direct transfer). It might seem that a reservoir of RNA-bound protein directly adjacent to chromatin DNA could increase chromatin occupancy by the protein. However, our results imply that RNA–nucleosome proximity without direct transfer should be insufficient for RNA-mediated facilitation of protein activity (*SI Appendix*, Fig. S6), though we acknowledge that our simulations only incorporate the α parameter in the presence of direct transfer. To test this question in a manner that avoids use of the α parameter, we employed single-molecule dynamics (SMD) simulations of mutually exclusive protein binding to RNA and nucleosomes tethered together. Our findings indicate that increasing RNA binding affinity increases RNA occupancy (*SI Appendix*, Fig. S11*A*) as expected, but slightly decreases nucleosome occupancy (*SI Appendix*, Fig. S11*B*). Similarly, the simulations show increased intermolecular distance between nucleosomes and nearby unbound protein (*SI Appendix*, Fig. S11*C*) and a reduced concentration of unbound protein in nucleosome-adjacent solvent space (*SI Appendix*, Fig. S11*D*), confirming an antagonistic relationship between RNA and nucleosome occupancy. We note that our SMD approach is a first approximation that does not, for example, account for Debye–Hückel effects on short range electrostatics, which could affect the assumption of isotropic diffusion around RNA/nucleosome. Thus, our collective findings suggest that RNA–nucleosome proximity alone is not sufficient for RNA-mediated recruitment of mutually exclusive binders to chromatin, with the limitation that more fine-grained interrogations may show otherwise under specific conditions.

## Discussion

### Implications for PRC2 Biology.

**Fig. 6.**
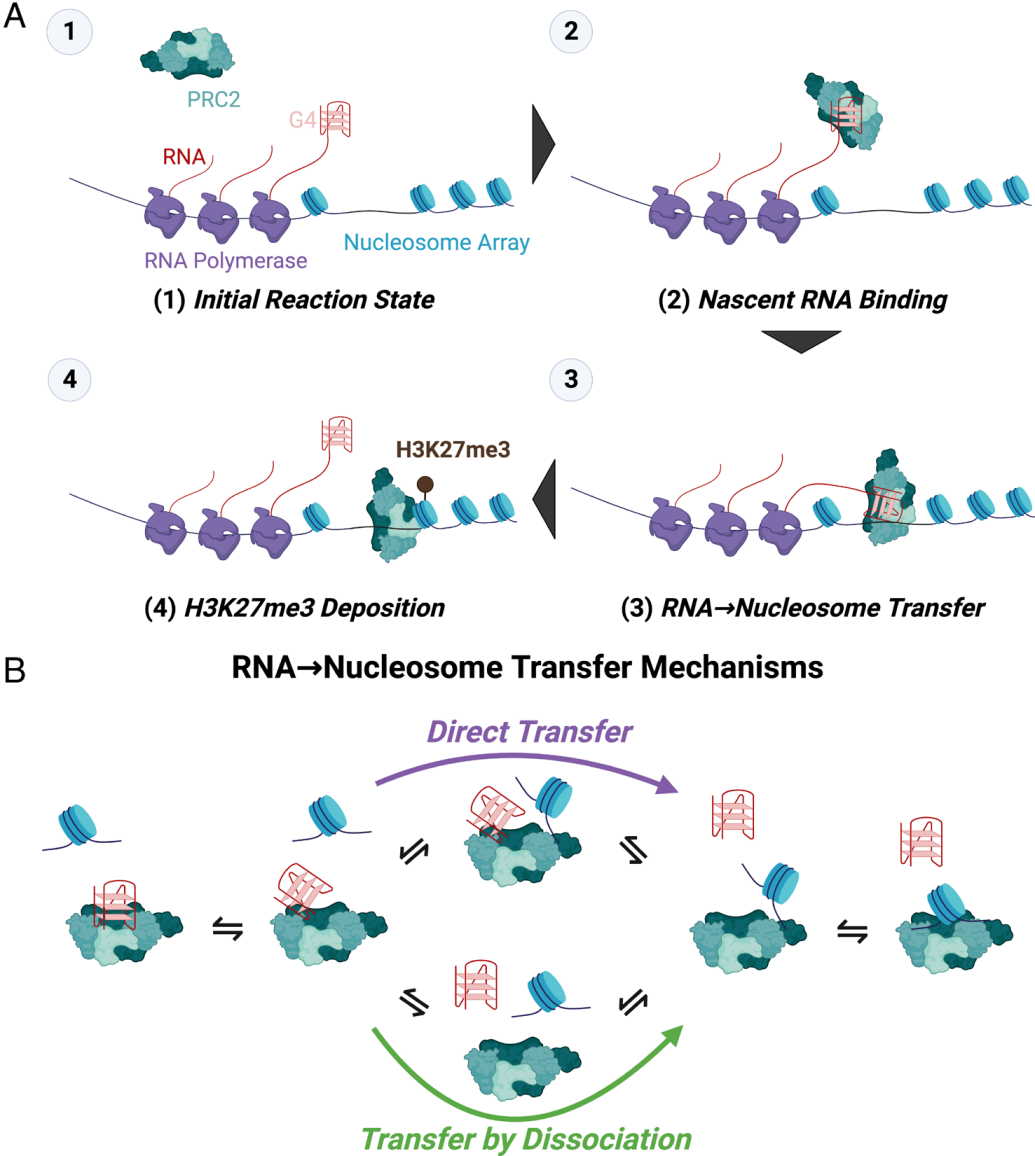
A direct transfer model of RNA regulation of PRC2 HMTase activity. (*A*) Proposed Steps for an RNA Recruitment Model of PRC2. (1) G4-containing nascent RNA at transcriptionally active PRC2 target genes (2) is bound by PRC2, (3) RNA-tethered PRC2 is transferred onto spatially proximal nucleosomes, then (4) PRC2 deposits its H3K27me3 mark. (*B*) Proposed Mechanisms for the Direct Transfer Step. PRC2 could have shared contacts for G4 RNA and nucleosome DNA binding but allow the ligands to occupy partially associated binding states that permit transient cobinding. Nucleosome DNA could give the appearance of actively disrupting a PRC2–RNA complex (*Left*) by forming a highly transient ternary intermediate where the PRC2-RNA interaction is destabilized (*Middle Top*). The unstable ternary intermediates may quickly dissociate to form a more stable PRC2-nucleosome (*Right*) or PRC2-RNA (*Left*) complex.

Our biophysical studies indicate that PRC2 is intrinsically capable of direct transfer between G4 RNA and nucleosome-linker-sized dsDNA (Fig. 2), and our computational investigations reveal that this behavior could allow RNA to have either an antagonistic or a synergistic effect on PRC2 activity depending on the relative RNA–nucleosome effective molarity (i.e., RNA–nucleosome proximity) (Fig. 4). These findings provide direct evidence for a mechanism (Fig. 6*A*) that theoretically allows RNA to facilitate PRC2 HMTase activity under certain conditions, which could reconcile prior perplexing in vitro and in vivo results where RNA was alternatively found to inhibit PRC2 or recruit it to sites of action. We propose that PRC2 binding to nascent RNA (Fig. 6*A*–1-2) could increase the effective molarity for direct transfer events (Fig. 6*A*–3), allowing increased chromatin association and H3K27me3 deposition (Fig. 6*A*–4) relative to an RNA-free (or RNA binding-free) system.

While these findings demonstrate that PRC2 direct transfer kinetics might support RNA-mediated recruitment to chromatin under certain conditions, they do not prove its occurrence in vivo. Specifically, we do not know what α parameter values pertain to physiological conditions, the effects of ligand/competitor length on direct transfer kinetics were not robustly explored here, and we only tested one combination of accessory proteins for the PRC2 complex. However, we note that our ligands represent the core G4 RNA structure and average nucleosome linker DNA length. Ideally, one would test in vivo a separation-of-function mutant that prevented direct transfer but retained full RNA and chromatin binding activities. However, we are pessimistic that such a mutant could be obtained, given that direct transfer is likely an intrinsic property of the nucleic acid-binding surfaces of PRC2. Although it is unclear whether direct transfer facilitates RNA-mediated PRC2 recruitment in vivo, we note that the ~1 mM nucleotide concentration of RNA in a human cell nucleus (43) could satisfy the 10 µM RNA competitor concentrations required for substantial direct transfer in our experiments, suggesting that PRC2 biology is impacted by flux through direct transfer.

The alternative situation, where RNA binding inhibits PRC2 activity, appears to occur in vivo. For example, it explains why many active genes have PRC2 close enough to be captured by ChIP (chromatin immunoprecipitation), yet the PRC2 does not act there (39). Furthermore, RNA inhibition of PRC2 has been shown in cells by Jenner et al. (20, 21). Consistent with these observations, our simulations (Fig. 4) indicate that RNA is indeed antagonistic below a certain threshold of α (i.e., low RNA–nucleosome proximity), although we have no way of predicting when and where these threshold conditions would be met in vivo. Importantly, our demonstration of PRC2’s direct transfer kinetics means that the recruitment and eviction models need not be mutually exclusive. As such, it is possible that RNA-mediated regulation of PRC2 operates as a “switch,” where predominantly free versus predominantly nascent RNA landscapes around target genes drive PRC2’s relationship with RNA being antagonistic versus synergistic, respectively (44).

### Biophysical Mechanism for Direct Transfer.

These findings demonstrate that PRC2 can translocate directly between polynucleotide species that are biologically relevant. Previous studies of such direct transfer have concerned homo-multimeric proteins, where a ligand bound to one protomer is in position to displace a second ligand bound to a nearby protomer. Therefore, the occurrence of direct transfer with PRC2 was unexpected, and it raises compelling questions about the underlying biophysical mechanism. We consider a model (Fig. 6*B*) that involves PRC2 ligands, such as RNA and nucleosome DNA, competing for the same or overlapping binding sites. Dynamic motion of the protein and/or the RNA gives a partially dissociated intermediate allowing the nucleosome to bind, forming an unstable ternary complex (Fig. 6*B*–top-middle). Full dissociation of the RNA ligand then allows full association of the nucleosome ligand (right).

We note that it is not yet clear how RNA and nucleosome DNA compete for binding on the surface(s) of PRC2, though some structural insights are emerging (45–47). This lack of definition in the PRC2 binding surfaces and the heterogeneity and complexity of PRC2 limit our ability to critically evaluate this model for PRC2 at this time. It is, for example, alternatively possible that the PRC2 direct transfer via an unstable ternary complex could be facilitated by distinct binding surfaces with mutual negative allosteric regulation. However, we believe several pieces of evidence favor the shared contacts model (Fig. 6*B*). First, prior work has implicated similar mechanisms in direct transfer kinetics, and suggested that many nucleic acid binding interfaces could have the capacity to support them (26, 35, 37). Indeed, our own concurrent studies (48) provide mechanistic evidence for direct transfer occurring commonly with nucleic acid binding proteins. Second, we also observed PRC2 direct transfer between identical (labeled versus unlabeled) G4 RNA species and between identical dsDNA species (Fig. 2). Unless PRC2 has multiple distinct binding sites with intersite negative allostery for each of these ligands, our observed direct transfer kinetics seem unlikely to be produced without shared contacts. Finally, recent structural and biophysical studies support some degree of overlap in PRC2 contacts with G4 RNA and dsDNA (47). Ultimately, however, elucidating the full-resolution mechanism that facilitates PRC2 direct transfer will require more structural and biophysical work.

### Implications for Other Chromatin Modifiers.

Accumulating evidence suggests that RNA-binding activity is common among chromatin-associated proteins (14, 49–52), and our simulations raise the possibility that direct transfer might be generally required for RNA-mediated recruitment models of such proteins. Our concurrent studies (48) suggest that the capacity for direct transfer could be quite common among other nucleic acid binding proteins. Furthermore, the k_θD_/k_−1P_ ratios for direct transfer reactions in Table 1 and our concurrent studies average ~10^5^ M^−1^, suggesting that flux through a direct transfer pathway (Fig. 6*B*) exceeds classic dissociation at competitor effective molarities above ~10 µM (48). We note, by the same k_θ_/k_−1_ metric, that the 10 µM competitor condition for direct transfer would also apply to proteins like SSB, CAP, and recA that are typically considered to be proficient at direct transfer (25–27). Micromolar effective molarities are likely to be achieved in cells, because the nucleotide concentration of RNA is ~1 mM (~50 µM of a 20-nt RNA) in a cell nucleus and DNA nucleotides are ~10- to 40-fold more concentrated (43, 53); thus, there is potential for biologically relevant flux through a direct transfer (versus classic) pathway in vivo. We note in the specific case of PRC2 that not all RNA in a cell nucleus would form the G4 RNA preferred by PRC2; however, only a minority of nuclear RNA must form a G4 structure to achieve micromolar G4 RNA concentrations, the effective molarity of G4s may be increased in some contexts (e.g., nascent RNA and nucleosome DNA), and PRC2 also exhibits intermediate affinity for many other RNA sequences/structures (16).

If direct transfer capability proves to be pervasive among chromatin-associated proteins, then our findings for PRC2 might explain why so many chromatin-associated proteins exhibit RNA-binding activity: intrinsic direct transfer capability could allow for RNA-mediated regulation. As a recent example, the RNA-binding domain of CCCTC-binding factor (CTCF) has been proposed to increase its search efficiency for DNA target sites [(54, 55); see also refs. 44 and 56]. In our work, it’s important to distinguish the characteristics of competitive binding (PRC2-like) (Fig. 4) versus independent binding (YY1-like) (Fig. 5) for direct transfer. In the former case, RNA can recruit protein under high RNA–nucleosome proximity conditions but actively antagonize protein activity under low RNA–nucleosome proximity conditions. In the latter case, while RNA could indeed recruit proteins if there is high RNA–nucleosome proximity, it may be unable to antagonize protein activity if RNA is predominantly in free solution, unless bound RNA affects other nucleosome binding-independent protein function(s). Thus, it is possible that chromatin-associated proteins could evolve PRC2-like versus YY1-like direct transfer in response to physiological pressures for tight regulation versus efficient recruitment, respectively. Future in vitro and in vivo studies with a diversity of chromatin-associated proteins are warranted to interrogate the prevalence and nature of direct transfer’s role(s) in RNA-mediated regulation of gene expression.

## Materials and Methods

### PRC2 Expression and Purification.

According to prior methodology (39), we used pFastBac vectors encoding N-terminally MBP-tagged fusions of each of the four core PRC2 subunits (EZH2, SUZ12, EED, and RBBP4), and either the embryonic (PRC2_5me_) or somatic (PRC2_5m_) isoform of AEBP2 (57), to prepare respective baculovirus stocks for co-infection of Sf9 cells. Then, according to prior methodology (16), cell paste containing expressed PRC2 was lysed, clarified, then purified by sequential amylose column chromatography, MBP-tag cleavage, heparin column chromatography, and size-exclusion column chromatography. The PRC2_5me_ protein was used for all reported experiments, except where otherwise indicated (Table 1).

### Preparation of Polynucleotides.

All oligos were ordered from IDT, and their sequences in IDT syntax are provided (*SI Appendix*, Table S1). For dsDNA constructs, complementary oligos ordered from IDT were mixed at 5 µM (ligand) or 300 µM (competitor) each in annealing buffer (50 mM TRIS pH 7.5 at 25 °C, 200 mM NaCl), subjected to a thermocycler program (95 °C for 10-min, 954 °C at 0.5 °C/min, hold at 4 °C) for annealing, then annealing confirmed via Native-PAGE. Concentrations of all ligands were confirmed spectroscopically using manufacturer-provided extinction coefficients.

### Binding Buffers.

All binding buffers (BB) contained 50 mM TRIS (pH 7.5 at 25 °C), 2.5 mM MgCl_2_, 0.1 mM ZnCl_2_, 0.1 mg/mL BSA, 5% v/v glycerol, and 2 mM 2-mercaptoethanol, plus a variable concentration of KCl (10, 25, 100, or 200 mM). Subscript of each binding buffer indicates the concentration of KCl in milli-molarity (e.g., BB_25_ = 25 mM KCl).

### FP-Based K_d_ Determination.

Pre-reaction mix was prepared with 5 nM ligand molecule in respective binding buffer (*Binding Buffers*), then dispensed in 36 µL volumes into the wells of a 384-well black microplate (Corning #3575). PRC2 was prepared at 10X the reported concentrations via serial dilution in binding buffer. Binding reactions were initiated by addition of 4 µL of PRC2 solution to the corresponding prereaction mix, then incubated 30 min at room temperature. Wells with binding buffer only were also included for blanking. Fluorescence polarization readings were then taken for 30 min in 30-s intervals with a TECAN Spark microplate reader (excitation wavelength = 481 ± 20 nm, emission wavelength = 526 ± 20 nm). Each experiment had two or four technical replicates per protein concentration (as indicated), and at least three independent experiments were performed per protein–polynucleotide combination.

Raw data were analyzed in R v4.1.1 with the FPalyze function (FPalyze v1.3.1 package; see *Data, Materials, and Software Availability*). Briefly, polarization versus time data were calculated for each reaction, the last 10 data points for each reaction averaged to generate an equilibrium polarization value, and then equilibrium polarization values were plotted as a function of protein concentration. Plot data were regressed with *SI Appendix*, Eq. **S2** to calculate K_d_^app^ for the interaction.

### FPCD Experiments.

Pre-reaction mix was prepared with 5 nM ligand molecule and PRC2 ≥ 2xK_dP_^app^ (at 25 °C) in binding buffer (see Binding Buffers), then dispensed in 36 µL volumes into the wells of a 384-well black microplate (Corning #3575). Decoy was prepared at 10X the reported concentrations via serial dilution in binding buffer or carrier polynucleotide (Table 1) at a concentration equal to the highest competitor concentration. Pre-reaction mix and competitor dilutions were then incubated at the indicated temperature to attain thermal and binding equilibrium (4 °C/90 min or 25 °C/30 min). Competitive dissociation reactions were initiated by addition of 4 µL of the respective competitor concentration to the corresponding pre-reaction mix, then fluorescence polarization readings were immediately (the delay between initiation of the first reactions and the first polarization reading was ~90 s) taken at 25 °C for 120 min in 30-s intervals with a TECAN Spark microplate reader (Ex = 481 ± 20 nm, Em = 526 ± 20 nm). Each experiment had 4 technical replicates per competitor concentration, and at least three independent experiments were performed per protein–polynucleotide combination unless otherwise indicated. All reported competition reactions used a PRC2 concentration of 100 nM.

Raw data were analyzed in R v4.1.1 with the FPalyze function (FPalyze v1.3.1 package). Briefly, polarization versus time data were calculated for each reaction, each reaction’s polarization data were normalized to the maximum and minimum polarization across all reactions, each normalized reaction was fit with an exponential dissociation function (*SI Appendix*, Eq. **S3.1**) to determine k_off_^obs^ (*SI Appendix*, Eq. **S3.2**), and k_off_^obs^ values were plotted as a function of competitor concentration. Plotted data (with background k_off_^obs^ subtracted to mitigate temperature effects on polarization) were regressed (the theoretical background for this approach is thoroughly covered in a separate manuscript) via *SI Appendix*, Eq. **S4.1** and then *SI Appendix*, Eq. **S4.2** with tuning parameters constrained to the *SI Appendix*, Eq. **S4.1** solutions, then the regression models compared with the Bayesian Information Criterion (58) (BIC). Rate constants (k_−1P_ and/or k_θD_) were determined from the best-performing regression model. If minimum polarization was not reached during competition experiments (e.g., due to a weak competitor), then it was manually defined with minimum polarization data from corresponding binding curve data (*FP-Based K_d_ Determination*).

### Ionic Strength Dependence.

Binding curve data (Fig. 3*A*) were regressed as described to determine K_d_^app^ (*FP-based K_d_ Determination*). Then, log_10_{K_d_^app^} versus log_10_{[KCl]} plots were regressed in R via stats:lm (package:function). Regression indicated m = 5.7 × 10^−4^ ± 0.34 (slope) and b = −8.5 ± 0.49 (intercept) for the G4 RNA data, and m = 1.4 ± 0.68 and b = −5.2 ± 1.1 for the dsDNA data, where values are the regressions’ estimate ± SE.

### PRC2 Reaction Scheme Simulations.

Reactions (Fig. 4*A*) were simulated and analyzed in R v4.1.1 with a custom script (*Data, Materials, and Software Availability*). Briefly, [E_T_], [N_T_], [R_T_], K_d_, k_−1_, k_θ_, k_cat_, and α were user-provided. Then, other rate constants and initial conditions were calculated via *SI Appendix*, Eq. **S7**, and the system of differential equations (*SI Appendix*, Eq. **S5**) was solved by numerical integration. Initial reaction rates (V_0_) were calculated as the average rate of change in [m_T_] during the first 5% of each reaction. By default, k_−1N_ = 9.1 × 10^−5^ s^−1^, k_−1R_ = 1.7 × 10^−3^ s^−1^, k_θN_ = 91, k_θR_ = 170, k_θNN_ = 260, K_dN_ = 5.1 nM, and K_dR_ = 2.3 nM were taken directly from Table 1 (k_−1_ are from self-competitions), k_cat_ = 10^−1^ s^−1^ was taken from PRC2 literature (41), [N_T_] = 5 nM was chosen arbitrarily, and all other parameter values were varied as indicated. By exception, k_θ_ = 0 for the *SI Appendix*, Fig. S6 studies, and k_−1R_ = 1.7 × 10^6^ s^−1^ and K_dR_ = 2.3 M for the *SI Appendix*, Fig. S5 studies. We note that the k_−1R_ value used was necessarily from BB_25_ buffer conditions, while other constants were from BB_10_ buffer conditions, but we also note that our salt dependency data (Fig. 3, Fig. 6, and Table 1) suggest that this produced no meaningful discrepancy.

### Cobinder Reaction Scheme Simulations.

Reactions (Fig. 5*A*) were simulated and analyzed in R v4.1.1 with a custom script (*Data, Materials, and Software Availability*). Briefly, [E_T_], [N_T_], [R_T_], K_d_, k_−1_, k_cat_, α, β, and δ were user-provided. Then, other rate constants and initial conditions were calculated via *SI Appendix*, Eq. **S7**, and the system of differential equations (*SI Appendix*, Eq. **S6**) was solved by numerical integration. Initial reaction rates (V_0_) were calculated as the average rate of change in [m_T_] during the first 5% of each reaction. By default, K_dR_ = 400 nM and K_dN_ = 200 nM were taken from Sigova et al. (42), k_1_ = 10^5^ M^−1^ s^−1^ was selected as a typical on-rate, k_−1_ = K_d_ × k_1_ s^−1^, k_cat_ = 10^−1^ s^−1^ was constrained to the value for PRC2, [N_T_] = 5 nM was used arbitrarily, δ = 1, and all other parameter values were varied as indicated. By exception, δ_1_ = 0 for the *SI Appendix*, Figs. S7 and S9*C* studies, δ_1_ = 10^−1^ for the *SI Appendix*, Figs. S9 *D*–*F* and S10 studies, δ_2_ = 10^2^ for the *SI Appendix*, Fig. S9*D* studies, δ_2R_ = 10^6^ and δ_2N_ = 10^4^ for the *SI Appendix*, Fig. S9*E* studies, δ_2R_ = 10^4^ and δ_2N_ = 10^6^ for the *SI Appendix*, Fig. S9*F* studies, δ_2_ = 10^5^ for the *SI Appendix*, Fig. S10 studies, and k_cat_ = 0 s^−1^ for the *SI Appendix*, Fig. S9 studies.

### Diagram, Reaction Scheme, and Figure Generation.

Diagrams were prepared with BioRender, reaction schemes were prepared with ChemDraw v21.0.0 (Perkin Elmer), tables were prepared with Word (Microsoft), graphs were prepared with R v4.1.1, protein structures were prepared in PyMOL v2.5.2 (Schrodinger), and figures were assembled in PowerPoint (Microsoft).

## Supplementary Material

Appendix 01 (PDF)

## Data Availability

GitHub hosts the FPalyze (github.com/whemphil/FPalyze) (59) R package. The custom scripts referenced in these methods are available on GitHub (github.com/whemphil/PRC2_Direct-Transfer_Manuscript) (60). pFastBac vectors for PRC2 expression have been deposited to AddGene (ID #125161-125165) by the Davidovich lab. All ligands and competitors are available from IDT via the sequences in *SI Appendix*, Table S2. Methodology on single-molecule simulations and equations can be found in *SI Appendix*, *Materials and Methods*. All other data are included in the manuscript and/or *SI Appendix*.
