# Supplementary material for: PRC2 direct transfer from G-quadruplex RNA to dsDNA has implications for RNA-binding chromatin modifiers
Source: Proc Natl Acad Sci U S A. 2023 May 30;120(23):e2220528120. doi: 10.1073/pnas.2220528120 (PMC10266057; doi:10.1073/pnas.2220528120)
Supplement: Supplementary file 1 — Appendix 01 (PDF) [file pnas.2220528120.sapp.pdf]

PNAS

Supporting Information for

PRC2 direct transfer from G-quadruplex RNA to dsDNA has implications for RNA-binding chromatin modifiers

Wayne O. Hemphill, Regan Fenske, Anne R. Gooding, Thomas R. Cech

Corresponding Author: Thomas R. Cech
Email: thomas.cech@colorado.edu

This PDF file includes:

Supporting text
Supp. Fig. 1-11
Supp. Tables 1-2

SUPPLEMENTARY MATERIALS & METHODS

Single-Molecule Dynamics Simulations:

Reactions (Supp. Fig. 11) were simulated and analyzed in R v4.1.1 with custom scripts (see Software, Data, and Materials Availability). Briefly, 'nucleosome' and 'protein' molecule coordinates were randomly scattered in a 3-dimensional simulation box with periodic boundary conditions, 'RNA' molecule coordinates were added linearly to both sides of each nucleosome in intervals of twice the molecular diameter (2x p_d), nucleosome and RNA coordinates were checked for molecular clash (proximity ≤ p_d), and coordinate generation was repeated if necessary. Then, molecular diffusion was approximated by 'random walk' changes in coordinates at each time-step, and inter-molecular binding was defined at the end of each time-step as a proximity of p_d or less between protein and nucleosome/RNA molecules. Molecules determined to be bound were set to a bound state, with their diffusion and additional binding capacity ablated, for a randomly sampled length of time based on the molecular pair's dissociation rate constant (k_{-1}).

Specifically, [E_T], [N_T], R_{nN} (RNA molecules per nucleosome), K_d, t (reaction time), τ (time-step), and ζ (dimensions of cubic simulation box) were user-provided, D (diffusion coefficient), p_d (molecular diameter), and A_n (Avogadro's number) were established constants, and k_1 and k_{-1} were calculated from other parameter values via Eq. 8.1-2. To generate initial conditions, numbers of nucleosome (N_n) and protein (E_n) molecules were calculated by rounding Eq. 8.3 to the nearest integers, numbers of RNA molecules (R_n) were calculated via Eq. 8.4, initial nucleosome and protein cartesian coordinates were sampled from a uniform distribution parameterized by [-ζ ÷2, ζ ÷2], RNA cartesian coordinates were calculated by 2x p_d-interval additions and subtractions to the nucleosome x-coordinates, an intermolecular distance of 2x p_d or greater was confirmed between all nucleosome and RNA molecules, and then coordinate assignment was repeated if necessary. To simulate diffusion between time-steps for each protein molecule in an unbound state, spherical coordinates for direction were sampled from a uniform distribution parameterized by [0, 2π], the spherical coordinate for magnitude was sampled from the Eq. 8.5 probability density function for random-walk diffusion, then spherical coordinates were converted to cartesian coordinates and added to the existing coordinate values. Molecules that diffused past a 'wall' in the defined simulation box during each time-step were moved a proportionate distance into the simulation box from the opposite 'wall' (periodic boundary conditions). To determine binding states after initial conditions and each diffusion step, the intermolecular distance to every protein molecule was calculated sequentially for every nucleosome/RNA molecule, the most proximal protein molecule with an intermolecular radius of p_d or less was identified (if any), a binding state value of zero (unbound) was confirmed for the protein and nucleosome/RNA molecules, a residence time was sampled from an exponential distribution parameterized by k_{-1} and rounded to the corresponding integer number of time-steps, and the time-step number set as the new binding state value. Zero binding states indicate unbound molecules, non-zero binding states indicate bound molecules, and non-zero binding state values drop by 1 at the end of each time-step (after diffusion and binding state updates).

Nucleosome/RNA occupancy was calculated via Eq. 8.6 as the fraction of nucleosome/RNA molecules in a protein-bound state, nucleosome-protein proximity was calculated via Eq. 8.7 as the average intermolecular distance between every nucleosome molecule and their closest R_{nN} ÷2 unbound protein molecules, and relative effective molarity was calculated via Eq. 8.8 as the relative concentrations of unbound protein in nucleosome-adjacent versus total solvent space. By default, t = 50 ms, τ = 10 ns, ζ = 1 μm, K_{dN} = 100 nM, K_{dR} = [1 M, 100 nM, 1 nM], [E_T] = 250 nM, [N_T] = 15 nM, R_{nN} = 8, D = 100 $\mu m^2\ s^{-1}$, p_d = 5 nm, and A_n = 6.022 $\times 10^{23}\ mol^{-1}$.

Equations:

For Eq. 1.1-10, rate constants are defined in Fig. 1 and Supp. Table 1, E is protein (PRC2), P is ligand, D is competitor, conjugations of these reactants are complexes, equations give rates of change for indicated reactants as a function of time (t), and bracketed terms indicate concentrations.

(Eq. 1.1) $[E]_t' = k_{-1P}[EP]_t + k_{-1D}[ED]_t - k_{1P}[E]_t[P]_t - k_{1D}[E]_t[D]_t$

(Eq. 1.2) $[P]_t' = k_{-1P}[EP]_t + k_{\theta D}[EP]_t[D]_t - k_{1P}[E]_t[P]_t - k_{\theta P}[ED]_t[P]_t$

(Eq. 1.3) $[D]_t' = k_{-1D}[ED]_t + k_{\theta P}[ED]_t[P]_t - k_{1D}[E]_t[D]_t - k_{\theta D}[EP]_t[D]_t$

(Eq. 1.4) $[EP]_t' = k_{1P}[E]_t[P]_t + k_{\theta P}[ED]_t[P]_t - k_{-1P}[EP]_t - k_{\theta D}[EP]_t[D]_t$

(Eq. 1.5) $[ED]_t' = k_{1D}[E]_t[D]_t + k_{\theta D}[EP]_t[D]_t - k_{-1D}[ED]_t - k_{\theta P}[ED]_t[P]_t$

(Eq. 1.6) $[E_T] = [E] + [EP] + [ED]$

(Eq. 1.7) $[P_T] = [P] + [EP]$

(Eq. 1.8) $[D_T] = [D] + [ED]$

(Eq. 1.9) $K_{dP} = \frac{k_{-1P}}{k_{1P}}$

(Eq. 1.10) $K_{dD} = \frac{k_{-1D}}{k_{1D}}$

For Eq. 2, P_E is polarization at equilibrium for a given $[E_T]$, P_{max} is the maximum polarization, P_{min} is the minimum polarization, $[E_T]$ is the total protein concentration, and K_d^{app} is the apparent dissociation constant.

(Eq. 2) $$P_E = (P_{max} - P_{min}) \frac{[E_T]}{[E_T] + K_d{}^{app}} + P_{min}$$

For Eq. 3.1-2, N_t is relative polarization at a given time (t), N_{min} is the minimum relative polarization, λ is the decay rate constant, and k_{off}^{obs} is the observed dissociation rate.

(Eq. 3.1) $$N_t = (1 - N_{min})\, e^{-\lambda t} + N_{min}$$
(Eq. 3.2) $$k_{off}{}^{obs} = (1 - N_{min})\, \lambda$$

For Eq. 4.1-2, apply Eq. 1 notation, and c_1 and c_2 are arbitrary tuning parameters. These equations are derived in concurrent studies (48).

(Eq. 4.1) $$\frac{-[EP]_0{}'}{[EP]_0} = k_{off}{}^{obs} \approx \frac{[D_T]^{c_2}}{c_1{}^{c_2} + [D_T]^{c_2}}\, k_{-1P} + k_{\theta D}\, [D_T]$$
(Eq. 4.2) $$\frac{-[EP]_0{}'}{[EP]_0} = k_{off}{}^{obs} \approx \frac{[D_T]^{c_2}}{c_1{}^{c_2} + [D_T]^{c_2}}\, k_{-1P}$$

For Eq. 5.1-11, terms are defined in Fig. 4a and Supp. Table 1, equations give rates of change for indicated reactants as a function of time (t), and bracketed terms indicate concentrations.

(Eq. 5.1) $[E]_t' = k_{-1N} ([EN]_t + [EN^m]_t) + k_{-1R} [ER]_t – [E]_t (k_{1N} ([N]_t + [N^m]_t) + k_{1R} [R]_t)$
(Eq. 5.2) $[N]_t' = [EN]_t (k_{-1N} + k_{\theta NN} [N^m]_t + \alpha k_{\theta R} [R]_t) – [N]_t (k_{1N} [E]_t + k_{\theta NN} [EN^m]_t + \alpha k_{\theta N} [ER]_t)$
(Eq. 5.3) $[R]_t' = [ER]_t (k_{-1R} + \alpha k_{\theta N} ([N]_t + [N^m]_t)) – [R]_t (k_{1R} [E]_t + \alpha k_{\theta R} ([EN]_t + [EN^m]_t))$
(Eq. 5.4) $[N^m]_t' = [EN^m]_t (k_{\theta NN} [N]_t + \alpha k_{\theta R} [R]_t + k_{-1N}) – [N^m]_t (k_{\theta NN} [EN]_t + k_{1N} [E]_t + \alpha k_{\theta N} [ER]_t)$
(Eq. 5.5) $[EN]_t' = [N]_t (k_{1N} [E]_t + k_{\theta NN} [EN^m]_t + \alpha k_{\theta N} [ER]_t) – [EN]_t (k_{cat} + k_{-1N} + k_{\theta NN} [N^m]_t + \alpha k_{\theta R} [R]_t)$
(Eq. 5.6) $[ER]_t' = [R]_t (k_{1R} [E]_t + \alpha k_{\theta R} ([EN]_t + [EN^m]_t)) – [ER]_t (\alpha k_{\theta N} ([N]_t + [N^m]_t) + k_{-1R})$
(Eq. 5.7) $[EN^m]_t' = k_{cat} [EN]_t + [N^m]_t (k_{\theta NN} [EN]_t + k_{1N} [E]_t + \alpha k_{\theta N} [ER]_t) – [EN^m]_t (k_{\theta NN} [N]_t + \alpha k_{\theta R} [R]_t + k_{-1N})$
(Eq. 5.8) $[E_T] = [E] + [EN] + [ER] + [EN^m]$
(Eq. 5.9) $[R_T] = [R] + [ER]$
(Eq. 5.10) $[N_T] = [N] + [N^m] + [EN] + [EN^m]$
(Eq. 5.11) $[m_T] = [N^m] + [EN^m]$

For Eq. 6.1-13, terms are defined in Fig. 5a and Supp. Table 1, equations give rates of change for indicated reactants as a function of time (t), and bracketed terms indicate concentrations.

(Eq. 6.1) $[E]_t' = k_{-1N} ([EN]_t + [EN^m]_t) + k_{-1R} [ER]_t – [E]_t (k_{1N} ([N]_t + [N^m]_t) + k_{1R} [R]_t)$
(Eq. 6.2) $[N]_t' = k_{-1N} ([EN]_t + \delta_{2N} [ENR]_t) – k_{1N} [N]_t ([E]_t + \alpha \delta_{1N} [ER]_t)$
(Eq. 6.3) $[R]_t' = k_{-1R} ([ER]_t + \delta_{2R} ([ENR]_t + [EN^mR]_t)) – k_{1R} [R]_t ([E]_t + \alpha \delta_{1R} ([EN]_t + [EN^m]_t))$
(Eq. 6.4) $[N^m]_t' = k_{-1N} ([EN^m]_t + \delta_{2N} [EN^mR]_t) – k_{1N} [N^m]_t ([E]_t + \alpha \delta_{1N} [ER]_t)$
(Eq. 6.5) $[EN]_t' = k_{1N} [E]_t [N]_t + \delta_{2R} k_{-1R} [ENR]_t – [EN]_t (k_{cat} + \alpha \delta_{1R} k_{1R} [R]_t + k_{-1N})$
(Eq. 6.6) $[ER]_t' = k_{1R} [E]_t [R]_t + \delta_{2N} k_{-1N} ([ENR]_t + [EN^mR]_t) – [ER]_t (k_{-1R} + \alpha \delta_{1N} k_{1N} ([N]_t + [N^m]_t))$
(Eq. 6.7) $[EN^m]_t' = k_{cat} [EN]_t + k_{1N} [E]_t [N^m]_t + \delta_{2R} k_{-1R} [EN^mR]_t – [EN^m]_t (k_{-1N} + \alpha \delta_{1R} k_{1R} [R]_t)$
(Eq. 6.8) $[ENR]_t' = \alpha (\delta_{1R} k_{1R} [EN]_t [R]_t + \delta_{1N} k_{1N} [ER]_t [N]_t) – [ENR]_t (\delta_{2R} k_{-1R} + \beta k_{cat} + \delta_{2N} k_{-1N})$
(Eq. 6.9) $[EN^mR]_t' = \beta k_{cat} [ENR]_t + \alpha (\delta_{1R} k_{1R} [EN^m]_t [R]_t + \delta_{1N} k_{1N} [ER]_t [N^m]_t) – [EN^mR]_t (\delta_{2R} k_{-1R} + \delta_{2N} k_{-1N})$
(Eq. 6.10) $[E_T] = [E] + [EN] + [ER] + [EN^m] + [ENR] + [EN^mR]$
(Eq. 6.11) $[R_T] = [R] + [ER] + [ENR] + [EN^mR]$
(Eq. 6.12) $[N_T] = [N] + [N^m] + [EN] + [EN^m] + [ENR] + [EN^mR]$
(Eq. 6.13) $[m_T] = [N^m] + [EN^m] + [EN^mR]$

For Eq. 7.1-5, apply Eq. 5 notation. For Eq. 7.5b, apply Eq. 6 notation.

(Eq. 7.1) $$k_1 = \frac{k_{-1}}{K_d}$$
(Eq. 7.2) $[E]_0 = [E_T]$
(Eq. 7.3) $[R]_0 = [R_T]$

(Eq. 7.4) $[N]_0 = [N_T]$
(Eq. 7.5a) $[N^m]_0 = [EN]_0 = [ER]_0 = [EN^m]_0 = 0$
(Eq. 7.5b) $[N^m]_0 = [EN]_0 = [ER]_0 = [EN^m]_0 = [ENR]_0 = [EN^mR]_0 = 0$

For Eq. 8.1-8, P(r | D, τ) is the relative likelihood function of displacement (r) given a time-step interval (τ) and diffusion coefficient (D), N_n is the number of nucleosome molecules, A_n is Avogadro's number, $[N_T]$ is the total concentration of nucleosome, ζ is the dimension length of a cubic simulation box, R_n is the number of RNA molecules, N_n is the number of nucleosome molecules, R_{nN} is the number of RNA molecules per nucleosome, $B_{N/R}$ is the fraction of nucleosome/RNA molecules bound by protein, N^{1+} is the number of protein-bound nucleosome molecules, R^{1+} is the number of protein-bound RNA molecules, P_{EN} is protein-nucleosome proximity, $\overrightarrow{N_\imath}$ is the position of the ith nucleosome molecule, $\overrightarrow{E_\imath{}^0{}_\jmath}$ is the position of the jth closest unbound protein molecule (to the ith nucleosome molecule), M_ε is relative effective molarity, p_d is molecular radius, E_ζ^0 is the number of unbound protein molecules in the simulation box, and E_i^0 is the number of unbound protein molecules within a 10 x p_d radius of the ith nucleosome molecule.

(Eq. 8.1) $k_1 = 8\,\pi\,D\,p_d\,A_n$
(Eq. 8.2) $k_{-1} = k_1\,K_d$
(Eq. 8.3) $N_n = A_n\,[N_T]\,\zeta^3$
(Eq. 8.4) $R_n = N_n\,R_{nN}$
(Eq. 8.5) $P(\vec{r} \mid D, \tau) = \frac{1}{\sqrt{12\,\pi\,D\,\tau}}\,e^{\frac{-r^2}{12\,D\,\tau}}$
(Eq. 8.6.1) $B_N = \frac{N^{1+}}{N_n}$
(Eq. 8.6.2) $B_R = \frac{R^{1+}}{R_n}$
(Eq. 8.7) $P_{EN} = \frac{1}{N_n}\sum_{i=1}^{N_n}\left(\frac{2}{R_{nN}}\sum_{j=1}^{\frac{R_{nN}}{2}}\left(\overrightarrow{N_\imath} - \overrightarrow{E_\imath{}^0{}_\jmath}\right)\right)$
(Eq. 8.8) $M_\varepsilon = \frac{3\,\zeta^3}{4\,\pi\,N_n\,(10\,p_d)^3\,E_\zeta^0}\sum_{i=1}^{N_n} E_i^0$

Glossary of Abbreviations and Model Parameters

Identifier	Description
PRC2	Polycomb Repressive Complex 2
YY1	Yin Yang 1 (transcription factor)
RBTF	RNA-binding Transcription Factor
FPCD	Fluorescence Polarization-based Competitive Dissociation (see Fig. 1)
k_{off}^{obs}	Apparent initial protein-ligand dissociation rate (see Fig. 1)
K_{dP}	Equilibrium dissociation constant for protein-ligand interaction (Table 1)
K_{dD}	Equilibrium dissociation constant for protein-competitor interaction
k_{-1P}	Unimolecular rate constant for intrinsic protein-ligand dissociation (Fig. 1)
k_{1P}	Bimolecular rate constant for association of protein and ligand (Fig. 1)
k_{-1D}	Unimolecular rate constant for intrinsic protein-competitor dissociation (Fig. 1)
k_{1D}	Bimolecular rate constant for association of protein and competitor (Fig. 1)
$k_{\theta P}$	Bimolecular rate constant for protein direct transfer from competitor to ligand (Fig. 1)
$k_{\theta D}$	Bimolecular rate constant for protein direct transfer from ligand to competitor (Fig. 1)
k_{-1N}	Unimolecular rate constant for intrinsic protein-nucleosome dissociation (Figs. 4-5)
k_{1N}	Bimolecular rate constant for association of protein and nucleosome (Figs. 4-5)
k_{-1R}	Unimolecular rate constant for intrinsic protein-RNA dissociation (Figs. 4-5)
k_{1R}	Bimolecular rate constant for association of protein and RNA (Figs. 4-5)
k_{cat}	Unimolecular rate constant for methyltransferase catalysis (Figs. 4-5)
$k_{\theta NN}$	Bimolecular rate constant for protein direct transfer from nucleosome to nucleosome (Fig. 4)
$k_{\theta N}$	Bimolecular rate constant for protein direct transfer from RNA to nucleosome (Fig. 4)
$k_{\theta R}$	Bimolecular rate constant for protein direct transfer from nucleosome to RNA (Fig. 4)
α	Tuning parameter for effective molarity of direct transfer reactions (Figs. 4-5)
β	Tuning parameter for the effect of co-bound RNA on methyltransferase catalysis by protein-nucleosome-RNA complex (Fig. 5)
δ_{1N}	Tuning parameter for the effect of pre-bound nucleosome on RNA association with protein-nucleosome complex (Fig. 5)
δ_{1R}	Tuning parameter for the effect of co-bound RNA on methyltransferase catalysis (Fig. 5)
δ_{2N}	Tuning parameter for the effect of co-bound RNA on nucleosome dissociation from protein-nucleosome-RNA complex (Fig. 5)
δ_{2R}	Tuning parameter for the effect of co-bound nucleosome on RNA dissociation from protein-nucleosome-RNA complex (Fig. 5)
E	Protein (Figs. 4-5)
N	Nucleosome (Figs. 4-5)
R	RNA (Figs. 4-5)
N^m	Methylated nucleosome (Figs. 4-5)
EN	Protein bound to nucleosome (Figs. 4-5)
ER	Protein bound to RNA (Figs. 4-5)
EN^m	Protein bound to methylated nucleosome (Figs. 4-5)
ENR	Protein bound to nucleosome and RNA (Fig. 5)
EN^mR	Protein bound to methylated nucleosome and RNA (Fig. 5)
H3K27me3	Concentration of methylated nucleosome product; i.e., $[m_T]$ from Eq. 5.11 & 7.13 (Figs. 4-5, Supp. Figs. 4-6)
RNA:Nuc<br>$[R_T]/[N_T]$<br>R:N	Ratio of RNA to nucleosome in reaction (Figs. 4-5, Supp. Figs. 4-10)

V_0	Initial rate of methyltransferase reaction (Figs. 4-5, Supp. Figs. 7-10)
K_{dN}	Equilibrium dissociation constant for protein-nucleosome interaction (Supp. Fig. 11)
K_{dR}	Equilibrium dissociation constant for protein-RNA interaction (Supp. Fig. 11)
B_R	Fraction of RNA bound by protein (Supp. Fig. 11)
B_N	Fraction of nucleosome bound by protein (Supp. Fig. 11)
P_{EN}	Metric for nucleosome proximity to unbound protein; Eq. 8.7 (Supp. Fig. 11)
M_ε	Metric for relative molarity of unbound protein in nucleosome-adjacent versus total solvent space; Eq. 8.8 (Supp. Fig. 11)

Supp. Table 1. Descriptions of various abbreviations and model parameters used in these studies.

Oligos Synthesized

Identifier		Sequence (IDT Nomenclature, 5' → 3')
r(GGAA)$_{10}$[A488]		rGrGrArArGrGrArArGrGrArArGrGrArArGrGrArArGrGrArArGrGrArArGrGrArArGrGrArArGr GrArA/3Alex488N/
r(GGAA)$_{10}$		rGrGrArArGrGrArArGrGrArArGrGrArArGrGrArArGrGrArArGrGrArArGrGrArArGrGrArArGr GrArA
r(G$_3$A$_2$)$_4$[F]		rGrGrGrArArGrGrGrArArGrGrGrArArGrGrGrArA/36-FAM/
r(G$_3$A$_2$)$_4$[A488]		rGrGrGrArArGrGrGrArArGrGrGrArArGrGrGrArA/3AlexF488N/
r(G$_3$A$_2$)$_4$		rGrGrGrArArGrGrGrArArGrGrGrArArGrGrGrArA
ds-d(N)$_{50}$[F]	s1	CGCATCGCATCGCATCGCATCGCATCGCATCGCATCGCATCGCATCGCAT/36-FAM/
	s2	ATGCGATGCGATGCGATGCGATGCGATGCGATGCGATGCGATGCGATGCG
ds-d(N)$_{50}$	s1	CGCATCGCATCGCATCGCATCGCATCGCATCGCATCGCATCGCATCGCAT
	s2	ATGCGATGCGATGCGATGCGATGCGATGCGATGCGATGCGATGCGATGCG
ds-[F]d(N)$_{60}$	s1	/56-FAM/GAAGTGCCCGTGACGCGCGCGACGCCAGCCGACGAAGGCGGGACCCAG AGCGCGCGCCGT
	s2	/56-FAM/ACGGCGCGCGCTCTGGGTCCCGCCTTCGTCGGCTGGCGTCGCGCGCGT CACGGGCACTTC
ds-d(N)$_{60}$	s1	GAAGTGCCCGTGACGCGCGCGACGCCAGCCGACGAAGGCGGGACCCAGAGCGCG CGCCGT
	s2	ACGGCGCGCGCTCTGGGTCCCGCCTTCGTCGGCTGGCGTCGCGCGCGTCACGGG CACTTC
r(A)$_{20}$		rArArArArArArArArArArArArArArArArArArArA

Supp. Table 2. Identities of Synthetic Polynucleotide Species. The nomenclature used for ordering oligos from IDT (Sequence) is provided for all oligos named (Identifier) in these studies.

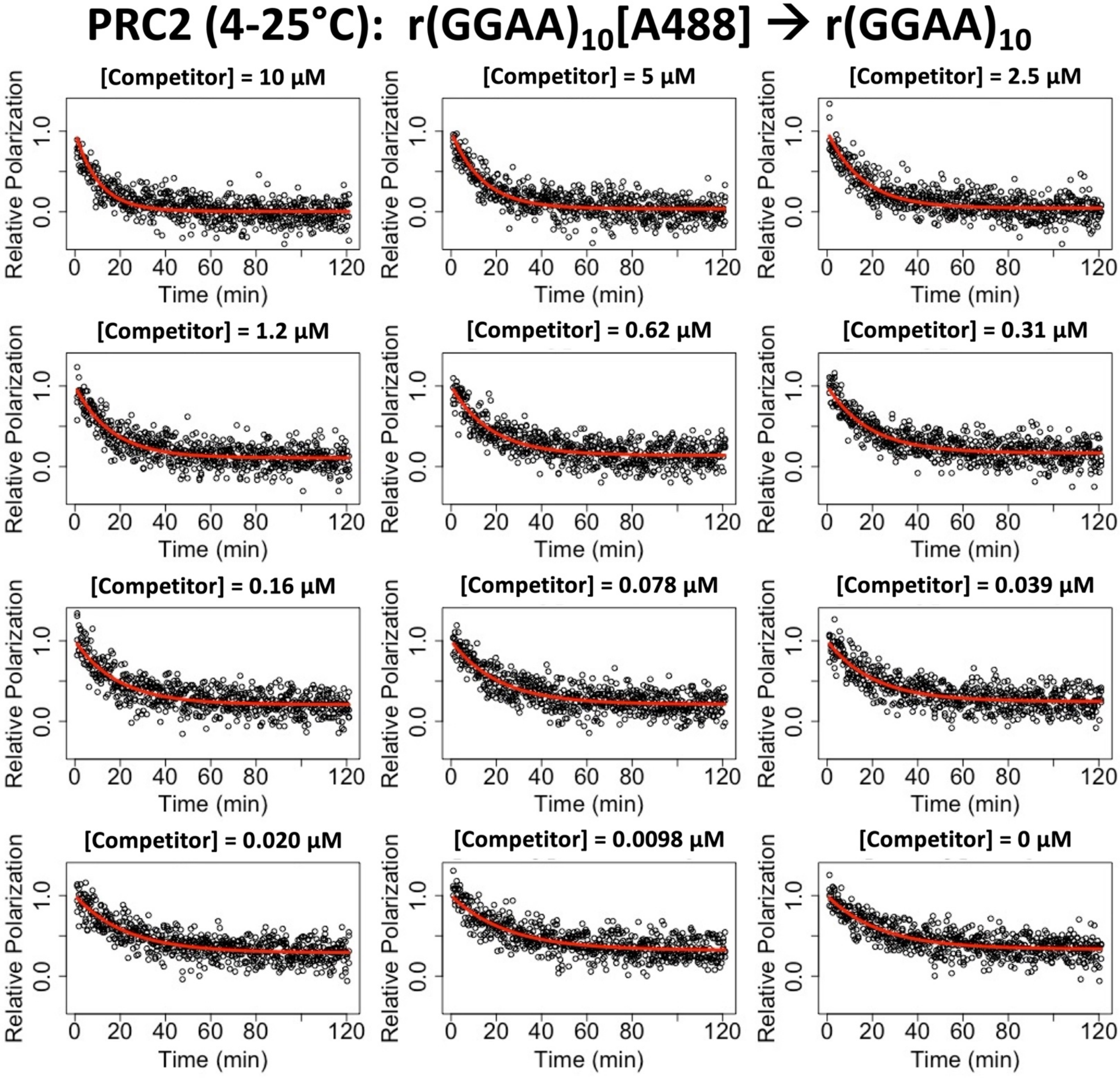

Supp. Fig. 1. Raw Data for Figure 2a. FPCD experiments (Fig. 1) were performed as described to replicate the original Wang et al and Long et al experiments over a range of competitor RNA concentrations. Raw data is from the same single representative experiment (of n = 3) as Fig. 2a, with four technical replicates. Reaction data for each competitor concentration is regressed with an exponential dissociation equation (Eq. 3.1), and the fit lines are shown (red lines). Polynucleotide species definitions are in Supp. Table 2.

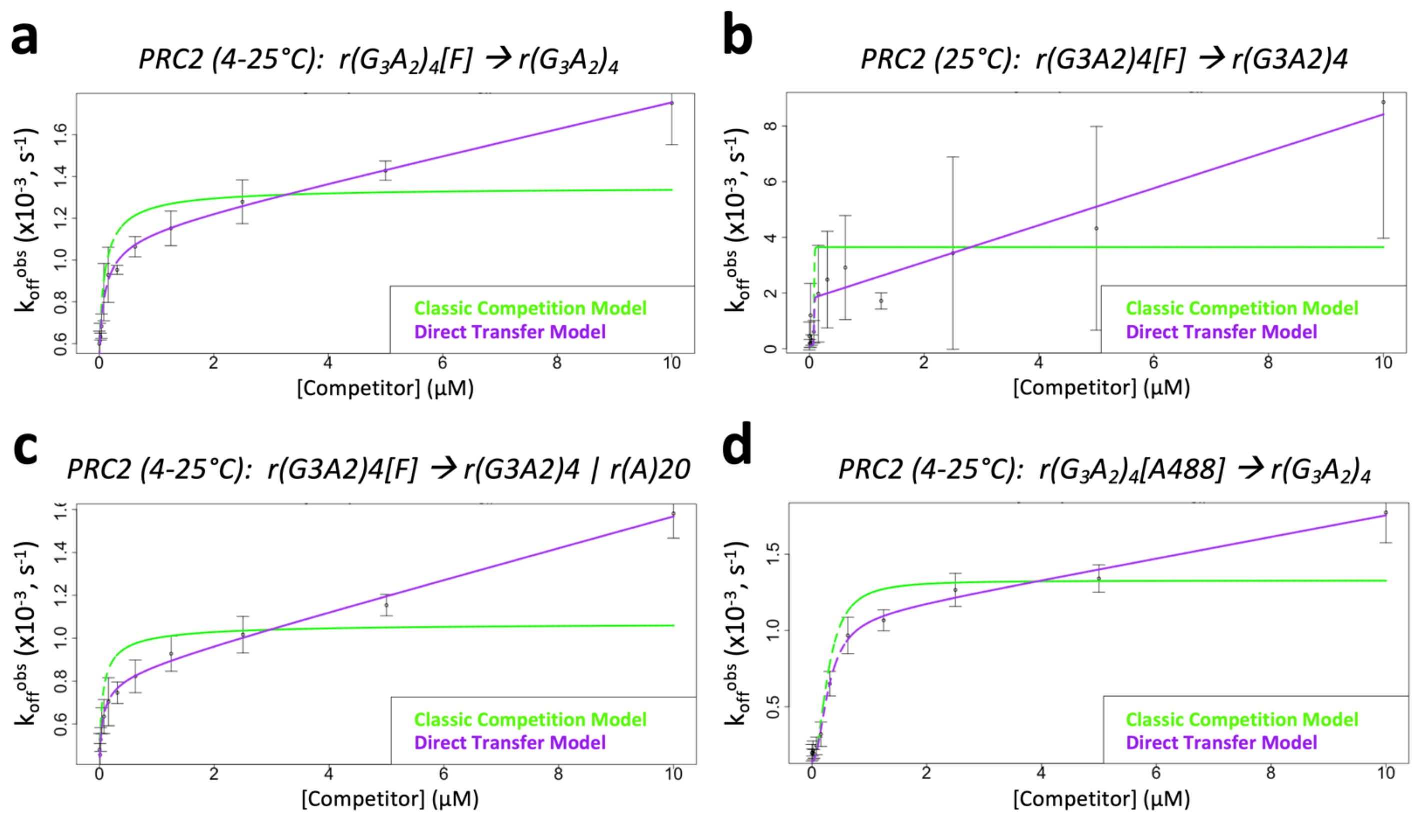

Supp. Fig. 2. A Simpler G4 RNA Exhibits Direct Transfer Kinetics Independent of Temperature, Polynucleotide Concentration, and Fluorescent Label. FPCD experiments (see Fig. 1) were performed (buffer = BB_{25}) as described for a simplified G-quad RNA as ligand and competitor. Data are from representative experiments (of n = 3), where error bars indicate mean ± SD for four technical replicates. Rate constant values from regression can be found in Table 1, additional nomenclature definitions are in Supp. Table 1, and polynucleotide species definitions are in Supp. Table 2. **[a]** Standard FPCD experiment. **[b]** FPCD experiment at constant 25°C – control for variable temperature artifacts. **[c]** FPCD experiment with nonbinding carrier RNA to keep total RNA concentration constant – control for nonspecific RNA concentration-dependent artifacts. **[d]** Standard FPCD experiment with different fluorophore.

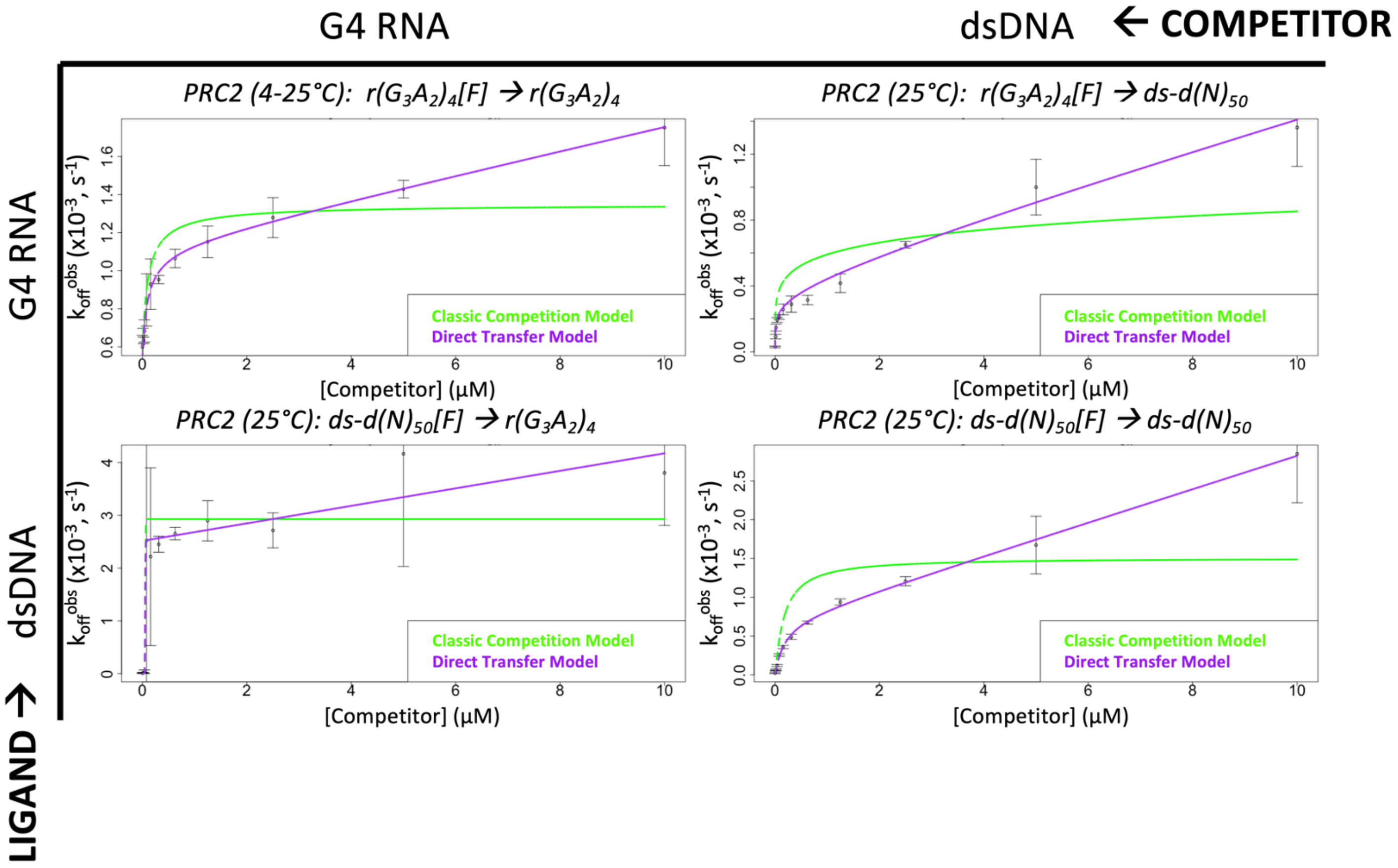

Supp. Fig. 3. PRC2 Exhibits Direct Transfer Kinetics for G4 RNA and Another dsDNA. FPCD experiments (Fig. 1) were performed (buffer = BB_{10}) as described for every ligand-competitor combination of a G-quad RNA and 50-bp dsDNA. Data are from representative experiments (of n = 3), where error bars indicate mean ± SD for four technical replicates. Rate constant values from regression can be found in Table 1, additional nomenclature definitions are in Supp. Table 1, and polynucleotide species definitions are in Supp. Table 2.

Product vs Time

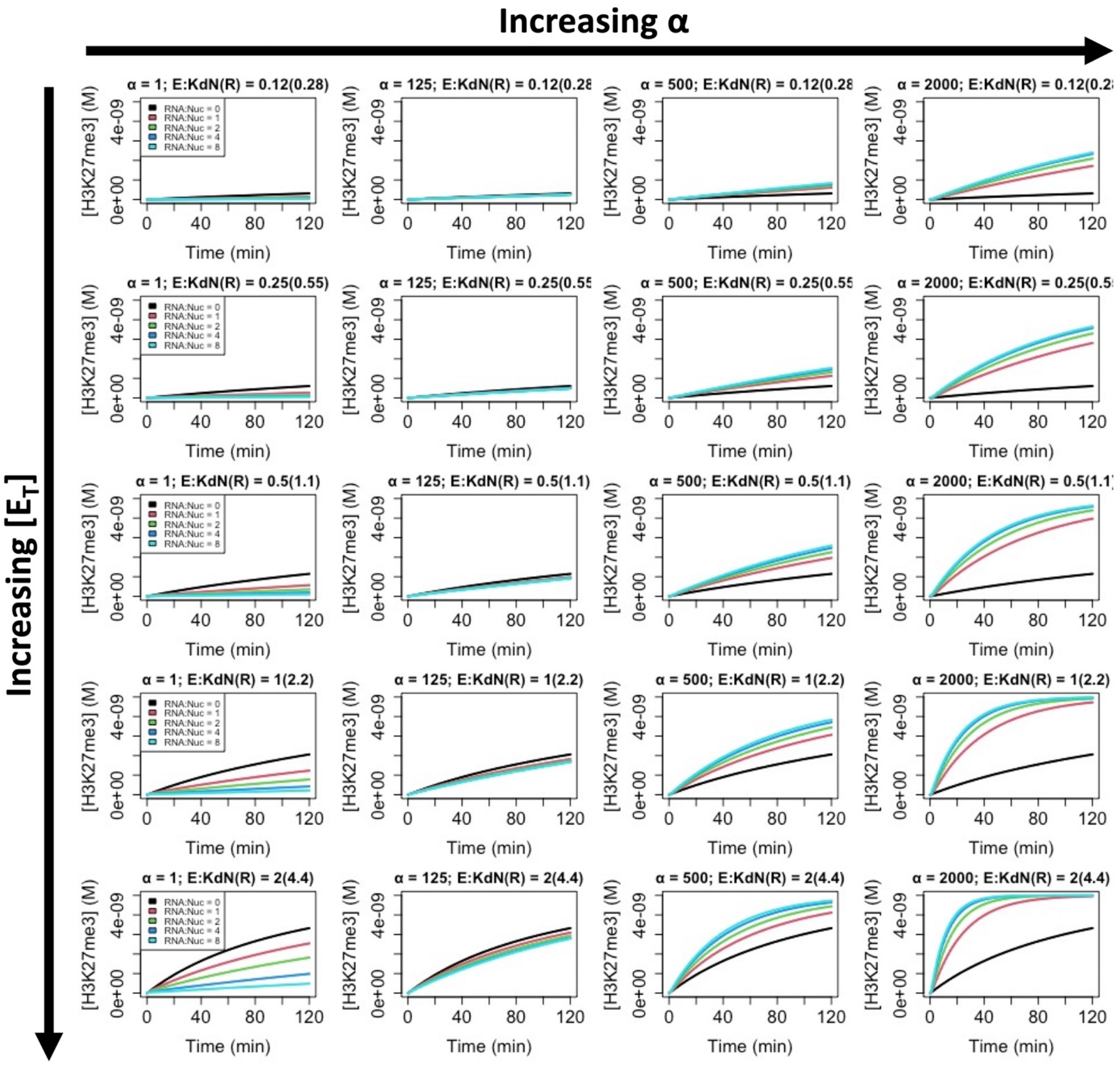

Supp. Fig. 4. Full Data Set for Fig. 4. Reactions monitor rate of nucleosome methylation (H3K27me3) over time under varying RNA-nucleosome molar ratios (RNA:Nuc), direct transfer effective molarity adjustments (α), and protein concentrations (E:Kd). Black curves represent HMTase time-course reactions in the absence of RNA, and the colored lines represent the effect of increasing RNA concentrations. Specific nomenclature definitions are in Supp. Table. 1, and explicit parameter values are provided in Materials & Methods.

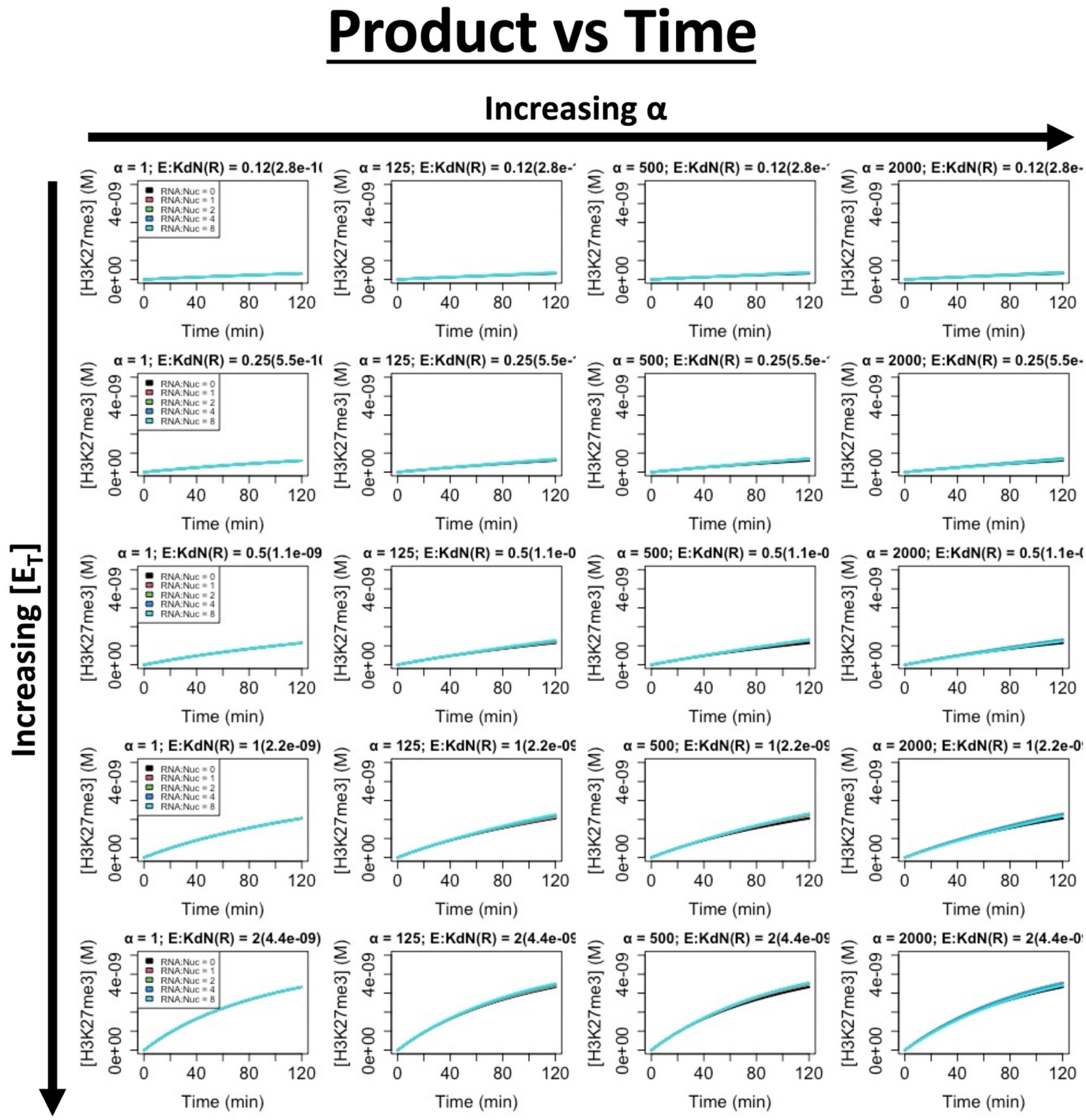

Supp. Fig. 5. Unstable RNA Binding Ablates RNA-Dependent Effects on PRC2 HMTase Activity. Fig. 4 simulations were altered to make RNA binding unstable (k_{-1R} = 1.7x10^6 s^{-1}, K_{dR} = 2.3 M). Reactions monitor rate of nucleosome methylation (H3K27me3) over time under varying RNA-nucleosome molar ratios (RNA:Nuc), direct transfer effective molarity adjustments (α), and protein concentrations (E:Kd). Black curves represent HMTase time-course reactions in the absence of RNA, and the colored lines represent the effect of increasing RNA concentrations. Specific nomenclature definitions are in Supp. Table. 1, and explicit parameter values are provided in Materials & Methods.

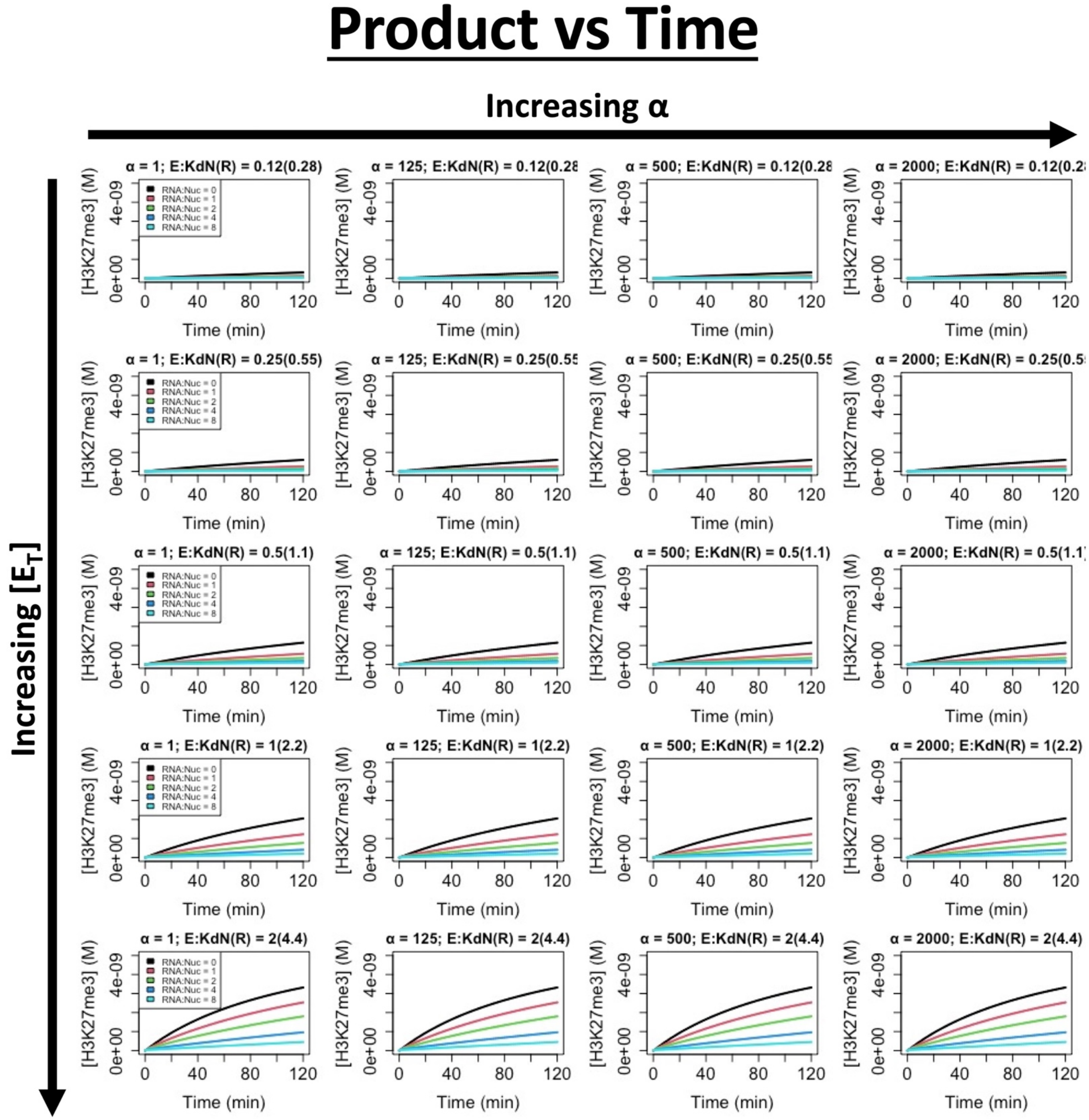

Supp. Fig. 6. PRC2's Proposed RNA-Dependent HMTase Boost is Dependent on Direct Transfer. Fig. 4 simulations were altered to eliminate direct transfer ($k_\theta = 0$). Reactions monitor rate of nucleosome methylation (H3K27me3) over time under varying RNA-nucleosome molar ratios (RNA:Nuc), direct transfer effective molarity adjustments (α), and protein concentrations (E:Kd). Black curves represent HMTase time-course reactions in the absence of RNA, and the colored lines represent the effect of increasing RNA concentrations. Specific nomenclature definitions are in Supp. Table. 1, and explicit parameter values are provided in Materials & Methods.

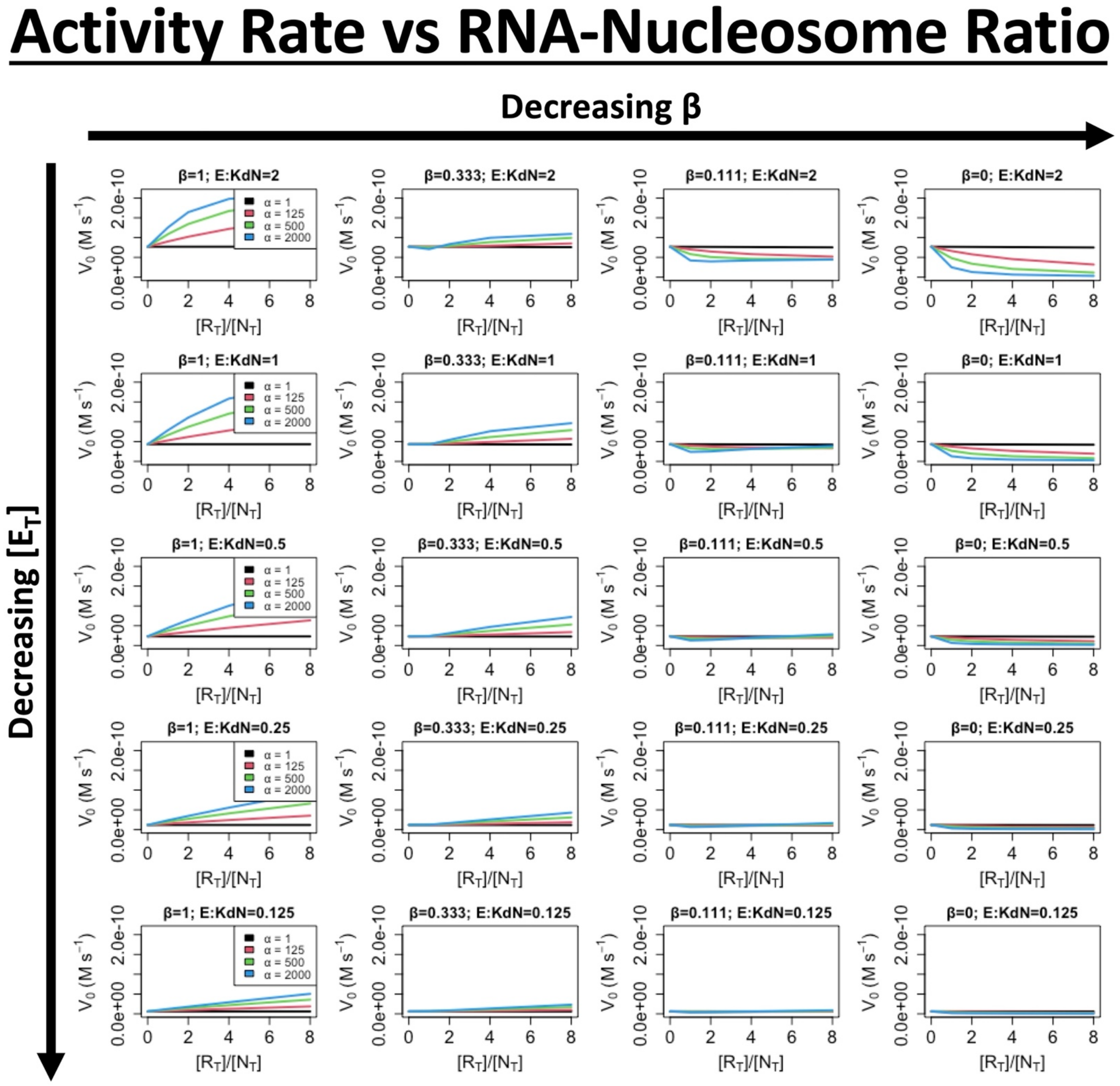

Supp. Fig. 7. Full Data Set for Fig. 5. Reactions monitor initial rate of nucleosome methylation (V_0) under varying RNA-nucleosome molar ratios ($[R_T]/[N_T]$), effective molarity adjustments (α), levels of RNA-mediated suppression of catalysis (β), and protein concentrations (E:Kd). Black curves represent the relationship between activity rate and RNA concentration when all reactants are in free solution (α = 1), and the colored lines represent the effect of increasing effective molarity for ternary complex-forming reactions (α > 1). Specific nomenclature definitions are in Supp. Table. 1, and explicit parameter values are provided in Materials & Methods.

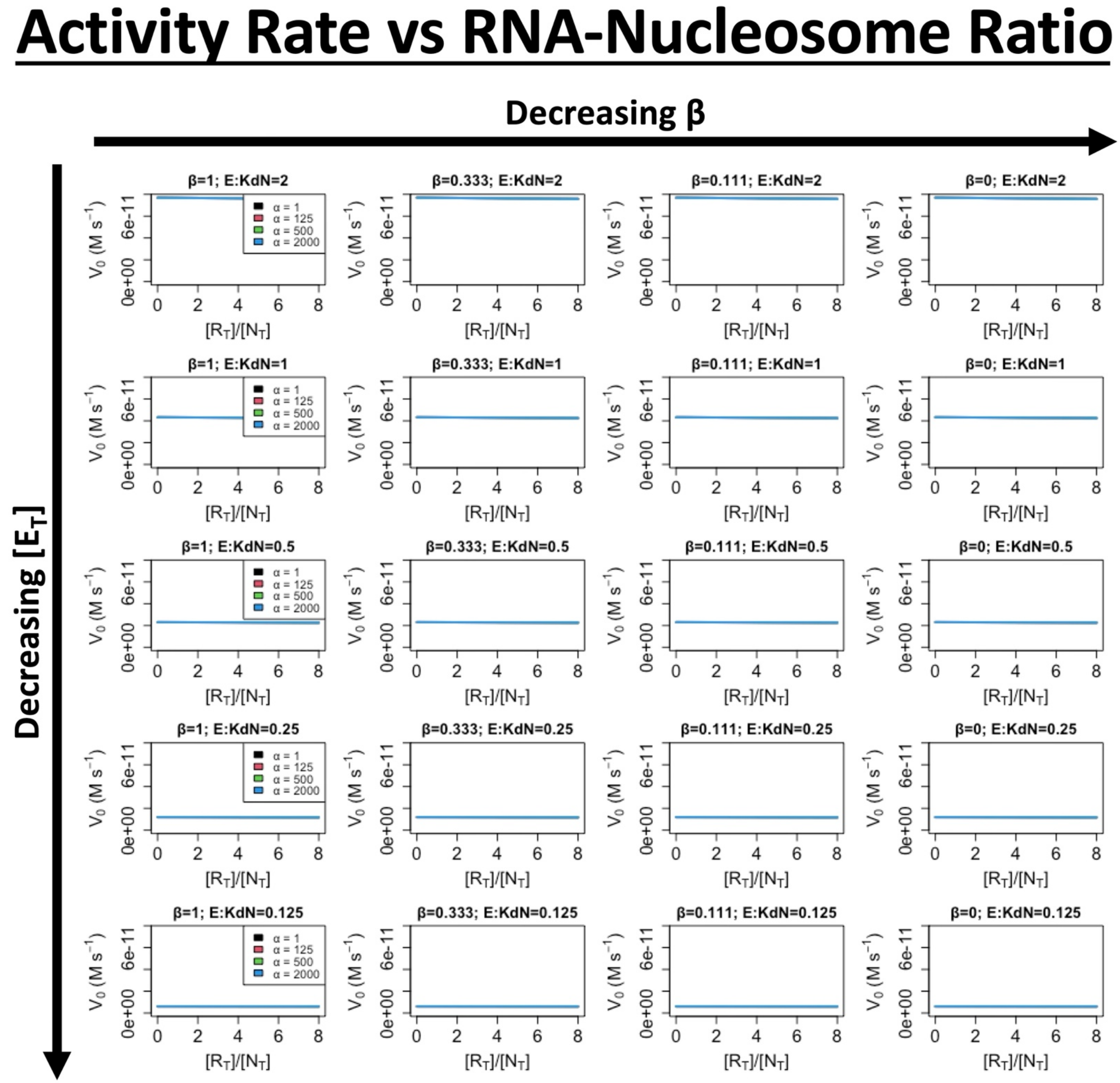

Supp. Fig. 8. RNA-mediated Activity Boosting for Co-Binders is Dependent on Ternary Complex Formation. Fig. 5 simulations were altered to prevent ternary complex formation ($\delta_1 = 0$). Reactions monitor initial rate of nucleosome methylation (V_0) under varying RNA-nucleosome molar ratios ($[R_T]/[N_T]$), effective molarity adjustments (α), levels of RNA-mediated suppression of catalysis (β), and protein concentrations (E:Kd). Black curves represent the relationship between activity rate and RNA concentration when all reactants are in free solution (α = 1), and the colored lines represent the effect of increasing effective molarity for ternary complex-forming reactions (α > 1). Specific nomenclature definitions are in Supp. Table. 1, and explicit parameter values are provided in Materials & Methods.

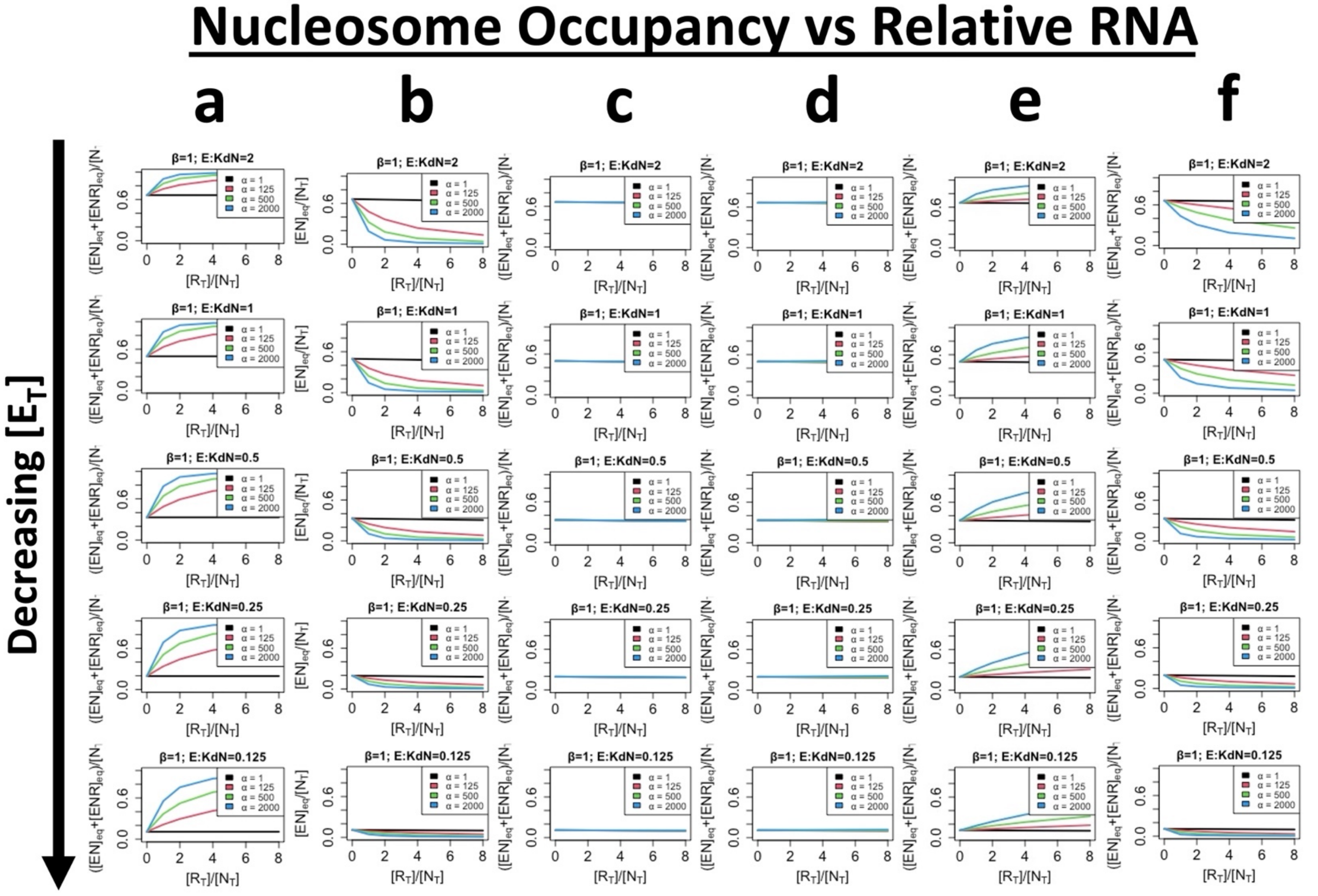

Supp. Fig. 9. Trends in RNA-mediated Function for Non-Catalytic Co-Binders Share Some Features of HMTase Co-Binders. The Fig. 5 reaction parameters were modified to disallow HMTase activity ($k_{cat} = 0$); any additional modifications are noted for each panel. Reactions monitor equilibrium nucleosome occupancy (defined for each panel) by protein (with β=1) under varying RNA-nucleosome molar ratios ($[R_T]/[N_T]$), effective molarity adjustments (α), and protein concentrations (E:Kd). Black curves represent the relationship between equilibrium binding and RNA concentration when all reactants are in free solution (α = 1), and the colored lines represent the effect of increasing effective molarity for ternary complex-forming reactions (α > 1). Specific nomenclature definitions are in Supp. Table. 1, and explicit parameter values are provided in Materials & Methods. **[a]** *Co-Binding Boosts Nucleosome Occupancy*. Reactions were performed as described, with nucleosome occupancy defined as the fraction of nucleosome in protein-nucleosome or protein-nucleosome-RNA complexes (i.e., protein can still perform its function when bound to RNA). **[b]** *RNA-mediated Suppression of Function During Nucleosome Occupancy Creates an Antagonistic RNA-Nucleosome Activity Relationship*. Reactions were performed as described, with nucleosome occupancy defined as the fraction of nucleosome in protein-nucleosome complexes only (i.e., protein cannot perform its function when bound to RNA). **[c]** *RNA-mediated Nucleosome Occupancy Boosts are Dependent on Ternary Complex Formation*. Reactions also had ternary complex formation ablated ($\delta_1 = 0$), with nucleosome occupancy defined like panel a. **[d]** Reactions also had the ternary complex destabilized without any ligand bias ($\delta_1 = 10^{-1}$, $\delta_2 = 10^{2}$), with nucleosome occupancy defined like panel a. **[e]** Reactions also had the ternary complex destabilized with a bias for RNA dissociation ($\delta_1 = 10^{-1}$, $\delta_{2N} = 10^{4}$, $\delta_{2R} = 10^{6}$), with nucleosome occupancy defined like panel a. **[f]** Reactions also had the ternary complex destabilized with a bias for RNA dissociation ($\delta_1 = 10^{-1}$, $\delta_{2N} = 10^{6}$, $\delta_{2R} = 10^{4}$), with nucleosome occupancy defined like panel a.

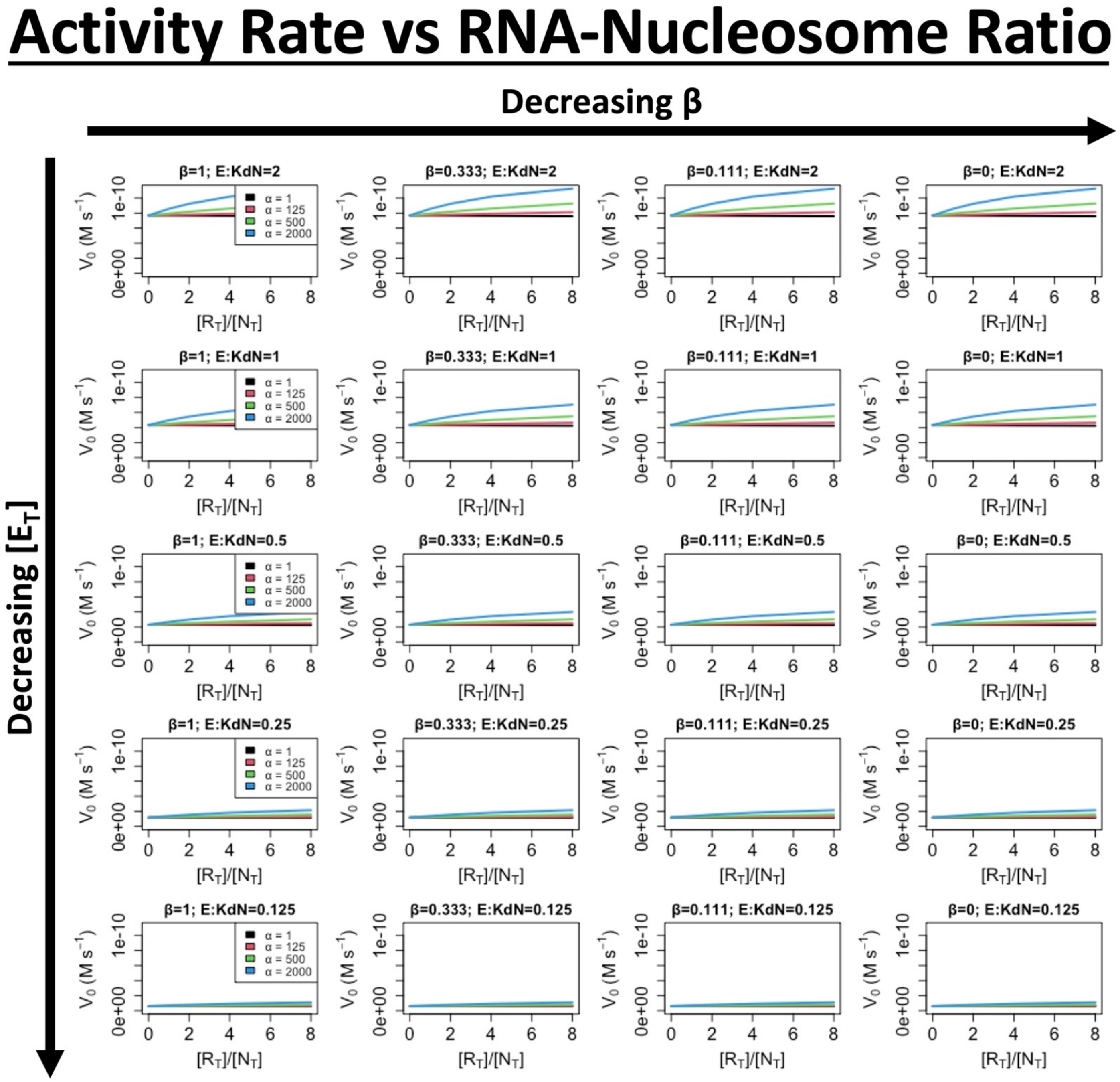

Supp. Fig. 10. An Unstable Ternary Complex is Sufficient for Some RNA-mediated Activity Boosting for HMTase Co-Binders. Fig. 5 simulations were altered to significantly destabilize the ternary complex ($\delta_1 = 10^{-1}$, $\delta_2 = 10^5$). Reactions monitor initial rate of nucleosome methylation (V_0) under varying RNA-nucleosome molar ratios ($[R_T]/[N_T]$), effective molarity adjustments (α), levels of RNA-mediated suppression of catalysis (β), and protein concentrations (E:Kd). Black curves represent the relationship between activity rate and RNA concentration when all reactants are in free solution (α = 1), and the colored lines represent the effect of increasing effective molarity for ternary complex-forming reactions ($\alpha > 1$). Specific nomenclature definitions are in Supp. Table. 1, and explicit parameter values are provided in Materials & Methods.

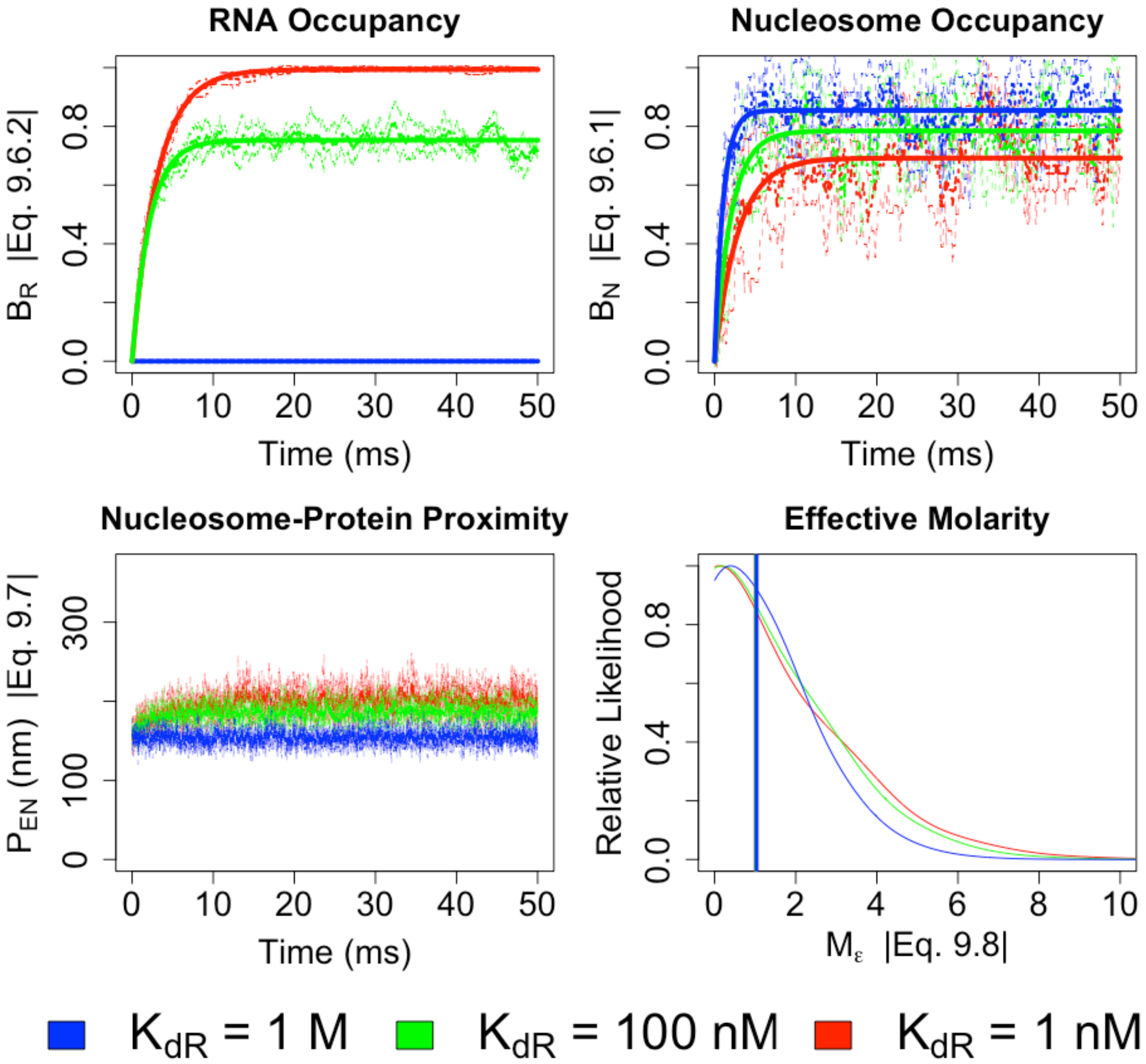

Supp. Fig. 11. RNA-Nucleosome Proximity Alone Doesn't Improve Nucleosome Occupancy for Mutually Exclusive Binders. Single-molecule dynamics simulations (millisecond timescale) were run with 250 nM protein, 100 nM nucleosome binding affinity, variable RNA binding affinities (K_{dR}), and mutually exclusive RNA/nucleosome binding, with an 8:1 RNA:nucleosome ratio with an RNA-nucleosome tethering length of twice the molecular diameter (5 nm). Colors correspond to different RNA binding affinities. Data are a composite of n = 4 replicate simulations. Specific nomenclature definitions are in Supp. Table. 1, and explicit parameter values are provided in Materials & Methods. **[a]** *Protein Association with RNA Over Time*. Dotted and dashed lines indicate mean ± SD, respectively, of the fraction of total RNA molecules bound (B_R; Eq 9.6.2). Solid lines indicate exponential association curve fit. **[b]** *Protein Association with Nucleosomes Over Time*. Dotted and dashed lines indicate mean ± SD, respectively, of the fraction of total nucleosome molecules bound (B_N; Eq 9.6.1). Solid lines indicate exponential association curve fit. **[c]** *Proximity of Nucleosomes to Nearest Protein Molecules Over Time*. The average intermolecular distance between nucleosome molecules and their four closest unbound protein molecules (P_{EN}; Eq 9.7) was calculated at every time point. Solid and dashed lines indicate

mean ± SD, respectively, of this average intermolecular distance. **[d]** *Relative Effective Molarity at Equilibrium*. The average concentration of unbound protein surrounding nucleosomes was divided by the concentration of unbound protein in the reaction to calculate relative effective molarity (M_ε; Eq 9.8) at every time point. Probability density plots of relative effective molarities across all equilibrium time points (time ≥ 10 ms) are shown, where vertical solid lines are means of respective probability density plots. Average equilibrium M_ε = [1.03, 1.02, 1.01] for K_{dR} = [1 M, 100 nM, 1 nM], respectively.
